# Midbrain organoids mimic early embryonic neurodevelopment and recapitulate LRRK2-p.Gly2019Ser-associated gene expression

**DOI:** 10.1016/j.ajhg.2021.12.009

**Published:** 2022-01-24

**Authors:** Alise Zagare, Kyriaki Barmpa, Semra Smajic, Lisa M. Smits, Kamil Grzyb, Anne Grünewald, Alexander Skupin, Sarah L. Nickels, Jens C. Schwamborn

**Affiliations:** 1 University of Luxembourg, Luxembourg Centre for Systems Biomedicine, 6, Avenue du Swing, L-4367 Belvaux, Luxembourg

**Keywords:** midbrain organoids, Parkinson disease, single-cell RNA sequencing, LRRK2-G2019S, neurodevelopment

## Abstract

Human brain organoid models that recapitulate the physiology and complexity of the human brain have a great potential for *in vitro* disease modeling, in particular for neurodegenerative diseases, such as Parkinson disease. In the present study, we compare single-cell RNA-sequencing data of human midbrain organoids to the developing human embryonic midbrain. We demonstrate that the *in vitro* model is comparable to its *in vivo* equivalents in terms of developmental path and cellular composition. Moreover, we investigate the potential of midbrain organoids for modeling early developmental changes in Parkinson disease. Therefore, we compare the single-cell RNA-sequencing data of healthy-individual-derived midbrain organoids to their isogenic LRRK2-p.Gly2019Ser-mutant counterparts. We show that the LRRK2 p.Gly2019Ser variant alters neurodevelopment, resulting in an untimely and incomplete differentiation with reduced cellular variability. Finally, we present four candidate genes, *APP*, *DNAJC6*, *GATA3*, and *PTN*, that might contribute to the LRRK2-p.Gly2019Ser-associated transcriptome changes that occur during early neurodevelopment.

## Graphical abstract

**Figure:**
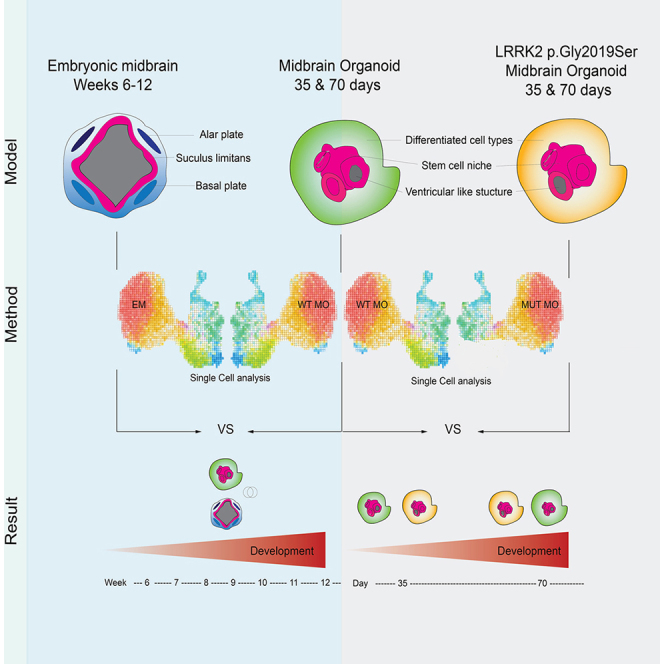

## Introduction

Parkinson disease (PD) is a multifactorial neurodegenerative disorder with varying motor and non-motor symptoms, characterized by the loss of dopaminergic neurons (DNs) in the substantia nigra pars compacta (SNpc) of the midbrain.^1^ The most common mutation associated with PD is c.6055G>A (p.Gly2019Ser) in leucine-rich repeat kinase 2 (*LRRK2*) (GenBank: NM_198578.4).^2^, ^3^, ^4^ LRRK2 is a multidomain protein involved in many cellular functions, including cell proliferation, survival regulation of neural stem cells (NSCs), and neurogenesis.^5^^,^^6^ Altered neurogenesis and neurodevelopment have been suggested to have major implications in the development of neurodegenerative diseases, including PD.^7^ Accordingly, various studies show an accelerated neuronal differentiation in *LRRK2*-mutant human cellular models, with a simultaneous impairment specifically of DN development.^8^, ^9^, ^10^ In particular, the interaction of LRRK2 with the canonical Wnt/b-catenin signaling pathway has been linked to the development of DNs through the regulation of axonal guidance, dendritic morphogenesis, and synapse formation.^11^, ^12^, ^13^, ^14^

Taking into consideration the complexity of the etiology of PD related to age, genetics, and environmental causes and the possibility of a neurodevelopmental component in PD, it is essential to have an adequate model, which can represent the human brain development and the manifestation of the disease. Studies on human postmortem brain tissue provided precious understanding of PD-associated alterations.^15^ However, postmortem tissues are generally available at the end stage of the disease and display a late stage in the disease progression. In order to overcome the limitation of understanding the disease development throughout life, we rely on various experimental models. Our understanding of pathological mechanisms underlying the disease largely depends on models that do not fully portray the complexity of the disease pathology or the cellular composition of the human brain. Genetic and toxin-based animal models often are not able to adequately capture the critical aspects of human PD, resulting in incomplete disease phenotypes.^16^ The discovery of induced pluripotent stem cells (iPSCs) and CRISPR-Cas9 technology surpassed this obstacle and enabled the access to human-derived cells for isogenic disease modeling.^17^, ^18^, ^19^ Although such 2D cultures capture the specific effect of mutation-induced PD and its molecular mechanisms, they still lack the cellular diversity of the human brain. To overcome these limitations, the recent developments in self-organizing 3D human-derived midbrain organoids represent a promising advancement in modeling neurodegenerative diseases.^9^^,^^20^, ^21^, ^22^, ^23^, ^24^

In order to study the role of human LRRK2 p.Gly2019Ser in a physiological context of early human development, we used previously published single-cell RNA-sequencing (scRNA-seq) datasets of human embryonic midbrain between developmental week 6 and week 11^25^ as well as healthy-individual-derived isogenic wild-type (WT) and LRRK2 p.Gly2019Ser midbrain organoids of 35 and 70 days of differentiation.^26^ We have previously demonstrated that the respective midbrain organoids comprise different neuronal types, including dopaminergic, GABAergic, glutamatergic, and serotonergic neurons as well as glia cells.^26^ First, we sought to use the single-cell transcriptomes of healthy midbrain organoids and the human embryonic midbrain to analyze the shared cellular identities and correlation between the *in vitro* and *in vivo* systems. Further, we exploit the transcriptome of the healthy and isogenic (in which LRRK2 p.Gly2019Ser has been inserted) midbrain organoids to investigate the LRRK2-p.Gly2019Ser-dependent changes in gene expression. We report that the midbrain organoids share proportionately similar transcriptomic profile and cell-type diversity with the developing human midbrain. Additionally, our analysis shows that midbrain organoids accurately adopt human midbrain development and are able to capture a LRRK2-p.Gly2019Ser-associated gene expression profile that might underlie *LRRK2*-mutation-related phenotypes.

## Material and methods

### Midbrain organoid generation from midbrain floorplate neural progenitor cells

Neural progenitor cells (NPCs) were derived from iPSCs of a healthy individual and isogenic LRRK2-p.Gly2019Ser-inserted cell line. Gene-editing of the iPSCs was done with CRISP-Cas9 and piggyBac systems, and it has been described in Qing et al., 2017.^27^ The derivation of NPCs from iPSCs and further organoid generation have been described in detail previously^9^^,^^26^ (Table S1). In brief, NPCs were cultured in N2B27 base medium supplemented with 2.5 μM SB-431542 (SB, Ascent Scientific), 100 nM LDN-193189 (LDN, Sigma), 3 μM CHIR99021 (CHIR, Axon Medchem), 200 μM ascorbic acid (AA, Sigma), and 0.5 μM SAG (Merck). For the derivation of midbrain, 3,000 NPCs were seeded per well in an ultra-low-attachment 96-well plate. For 7 days, cells were kept under maintenance conditions, following 3 days of pre-pattering where LDN and SB were withdrawn, and CHIR concentration was reduced to 0.7 μM. On day 9 of organoid culture, the differentiation was induced by changing the medium to N2B27 with 10 ng/mL brain-derived neurotrophic factor (BDNF, Peprotech), 10 ng/mL glial-cell-derived neurotrophic factor (GDNF, Peprotech), 200 μM AA, 500 μM dibutyryl cAMP (Sigma), 1 ng/mL TGF-β3 (Peprotech), 10 μM dual antiplatelet therapy (DAPT) (Cayman), and 2.5 ng/mL ActivinA (Peprotech). The organoids were cultured under static conditions with media changes every third day for 35 or 70 days. 30 midbrain organoids of each condition (WT35, WT70, MUT35, and MUT70) were pulled for Drop-seq analysis as described in Smits et al., 2020.^26^

### Immunofluorescence staining

Midbrain organoids were fixed with 4% paraformaldehyde (PFA) overnight at 4°C followed by three washes with PBS for 15 min. The washed organoids were embedded in 3%–4% low-melting point agarose in PBS. Embedded organoids were sectioned into 50 μm sections with vibratome (Leica VT1000s). Organoid sections were blocked with 0.5% Triton X-100, 0.1% sodium azide, 0.1% sodium citrate, 2% BSA, and 5% normal donkey serum in PBS for 90 min at room temperature (RT) on a shaker. We diluted the primary antibodies in the same solution but with 0.1% Triton X-100 instead. The sections were incubated with the primary antibodies for 48 h at 4°C. Next, they were washed three times with PBS and subsequently blocked for 30 min at RT on a shaker. Next, sections were incubated with the secondary antibodies diluted in 0.05% Tween-20 in PBS for 2 h at RT and subsequently washed twice with 0.05% Tween-20 in PBS and once with Milli-Q water before mounting them in Fluoromount-G mounting medium (Southern Biotech). The primary antibodies used were TH rabbit Abcam ab112, FOXA2 mouse Santa Cruz sc-101060, and EN1 goat Santa Cruz sc- 46101. The secondary antibodies used were Hoechst 33342 solution (20 mM) Invitrogen 62249, anti-rabbit secondary 488 Thermo Fisher a21206, anti-mouse secondary 568 Invitrogen A10037, and anti-goat secondary 647 Invitrogen A21447.

### Data pre-processing

In this study, we used already published scRNA-seq datasets. The midbrain organoids dataset was published from our lab,^26^ while the other three datasets (embryonic midbrain, embryonic prefrontal cortex, and cortical organoids) are external^25^^,^^28^^,^^29^ (Figure S1). scRNA-seq data from 30 pooled midbrain organoids per cell line and time point were generated following the Drop-seq pipeline.^30^ Reads were mapped to human reference genome hg38 (GRCh38.87). From midbrain organoids datasets, cells having unique feature counts over 2,500 were removed as probable doublets or multiplets. Similarly, low-quality cells or empty droplets were further filtered out with unique feature counts below 100 (for day 35 data) and 200 (for day 70 data) and mitochondrial transcripts above 30% (Figure S2). Embryonic midbrain scRNA-seq data did not include any mitochondrial (MT) genes, thus to make midbrain organoid data more comparable to the embryonic midbrain data, we removed all MT genes from midbrain organoid datasets after quality control (QC). After QC, WT35 midbrain organoids included 2,864 cells, WT70 included 2,005 cells, MUT35 included 2,946 cells, and MUT70 included 2,660 cells.

The external datasets of embryonic midbrain, prefrontal cortex, and cortex organoid did not show any outliers in terms of doublets or empty droplets. Therefore, no additional QC was applied to these datasets.

Embryonic midbrain data of developmental week 6 to 11 included in total 1,977 cells, embryonic prefrontal cortex data at developmental stages between gestational weeks 8 and 26 included 2,309 cells, and cortex organoid data from 1 month old organoid comprised 4,832 cells.

### Data integration and normalization

To better transmit the biological information between *in vivo* and *in vitro* ventral midbrain datasets, midbrain organoid data (WT35, WT70, MUT35, and MUT70) and embryonic midbrain data were integrated with the Seurat integration analysis workflow.^31^ Integration was performed on the basis of the top 20 dimensions. RNA assay data of integrated object were log normalized and scaled to 10,000 transcripts per cell.

### Cell type identification

After the integration of embryonic midbrain and midbrain organoid datasets, integrated object was scaled and principal-component analysis (PCA) was applied. Cell clustering was performed on the basis of the top 20 principal components via Louvain algorithm modularity optimization with a resolution of 0.5. Uniform manifold approximation and projection (UMAP) was used for cell cluster visualization.^32^ Nine distinct cell clusters were identified in the UMAP plot. Clusters 0 and 7 were present only in midbrain organoids and located in a close proximity to each other in the UMAP plot, indicating their high similarity and *in vitro* specificity. Because of this overclustering both clusters were pulled, resulting in eight distinct cellular identities labeled 1–8. For cell type identification, a binarized gene list across cell types from La Manno et al., 2016^25^ was used. This list of genes comprises information about the marker genes in a binarized manner, where 1 means that gene is marking a specific cell population and 0 means that it cannot be considered as a marker gene. For more details on how this list is generated, please refer to La Manno et al., 2016.^25^ Expression of each cluster-defining gene was overlapped with the marker gene (1) in the marker matrix from La Manno et al., 2016.^25^ The total number of marker genes of a particular cell type of La Manno et al., 2016^25^ that was present in each cluster of embryonic-midbrain- and midbrain-organoid-integrated dataset is visualized in Figure S4A. Cellular subtypes described by La Manno et al., 2016^25^ were grouped in five major neuronal identity clusters—neurons subdivided in dopaminergic neurons (DNs) and non-dopaminergic neurons (non-DNs), then neuroblasts (NBs), progenitors (PROGs), and radial glia cells (RGLs). In addition, we identified non-neuronal identity cell populations—pericytes and endothelial cells. Cell types were assigned on the basis of the highest number of major cluster marker genes being expressed in the respective clusters of integrated embryonic midbrain and midbrain organoid dataset.

### Differential gene expression analysis

Differentially expressed genes (DEGs) were detected with the FindMarkers function of the Seurat pipeline with the default thresholds. In all comparisons, we used the MUT midbrain organoids as ident.1 and the WT midbrain organoids as ident.2.

### Pathway analysis

Pathway enrichment analysis was performed with MetaCore version 21.1 build 70400 on the basis of DEGs detected with the FindMarkers function from Seurat. DEGs were filtered for fold change (FC) > 0.25 and p adj. value < 0.05. From the analysis, we obtained the most significant enriched pathways, GO processes, network processes, and related diseases lists. The most significantly enriched pathways were illustrated in GraphPad Prism 9.

### Cytoscape

Cell-cluster-specific genes were identified with the FindAllMarkers function from Seurat. The top 100 marker genes of each cell cluster were visualized in the network created with the Cytoscape software version 3.8.0.

### Pseudotime analysis

Pseudotime analysis was performed with the Monocle package version 3. Merged Seurat object was uploaded in the Monocle workflow. Cell clustering was performed on the basis of 150 principal components with default settings. UMAP was used for visualization. Because Monocle does not allow a full metadata integration from Seurat object, we assigned cell identities manually to correspond to the ones previously defined. For the comparison between developmental stages of embryonic midbrain and midbrain organoid, we used the align_cds function to remove the batch effect between *in vivo* and *in vitro* midbrain systems. As a starting point for cell ordering along the pseudotime trajectory, the NB *in vitro* cluster of WT35 was chosen. For the comparison between developmental stages of WT midbrain organoids and MUT midbrain organoids, the same starting point of the NB *in vitro* cluster of WT35 was chosen. Genes that vary the most over the pseudotime were computed with the fit_models function. Midbrain-organoid- and embryonic-midbrain-integrated Seurat object was subset by pseudotime genes for the visualization of their expression in midbrain organoids.

### Statistical analysis

If not stated otherwise, statistical analysis of scRNA-seq data was performed with RStudio R version 3.6.2 with the ggplot2 package. For all comparison, non-parametric Kruskal-Wallis test was performed. Statistical significance between comparisons are represented with asterisks: p < 0.05^∗^, p < 0.01^∗∗^, p < 0.001^∗∗∗^, p < 0.00001^∗∗∗∗^.

### *In vitro* and *in vivo* midbrain data comparison to the cortex

WT midbrain organoids and embryonic midbrain were merged with embryonic prefrontal cortex and integrated on the basis of the top 20 dimensions. SCTransform normalization was applied to reduce the technical variation in the data and stabilize gene abundance levels, which can be highly variable between *in vitro* and *in vivo* tissues, especially between different tissue types—midbrain and cortex.^33^ We determined mutual genes between midbrain organoids, embryonic midbrain, and embryonic prefrontal cortex by intersecting row names of respective datasets. Integrated object was subset by mutual genes. The top 2,000 variable genes in this subset of complete integrated dataset were detected with the FindVariableFeature function.

For the comparison of embryonic midbrain to cortical organoid, datasets were merged, SCTranformed, and subset by the mutual genes for the correlation analysis.

### Ethical approval

The responsible national ethical commission has approved the study under the CNER report no. 201901/01. Written informed consent was obtained from all individuals who donated samples to this study (Smits et al., 2020).^26^ The cell lines used in this study are summarized in Table S1.

## Results

### Midbrain organoids show a gene expression signature comparable to the human embryonic midbrain

To assess the similarity between the *in vitro* and *in vivo* midbrain systems, we compared scRNA-seq data of midbrain organoids cultured for 35 days (WT35) and 70 days (WT70)^26^ to the human embryonic midbrain of developmental weeks 6–11.^25^ In addition, to investigate possible transcriptome similarities between midbrain organoids and other brain regions during early development, we compared the scRNA-seq data of midbrain organoids to scRNA-seq data of the human embryonic prefrontal cortex.^28^

**Figure 1:**
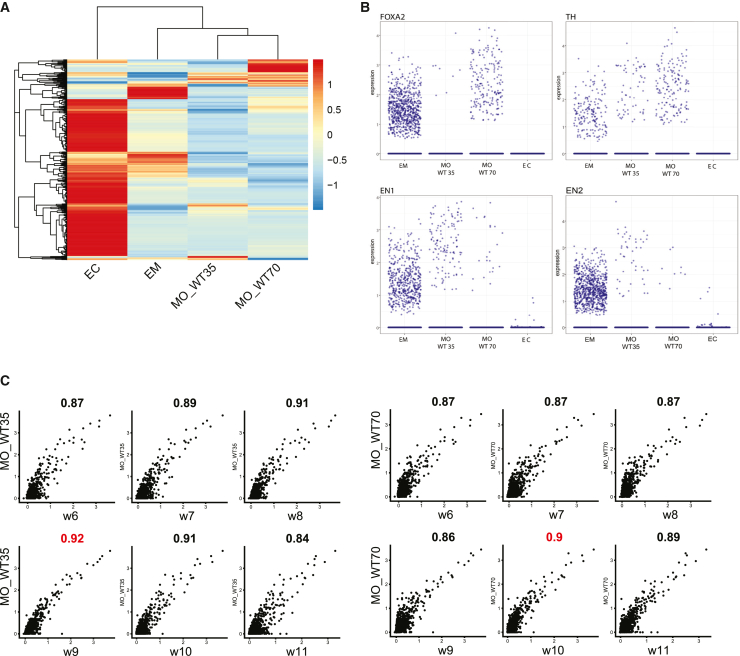
Midbrain organoids show a genetic signature comparable to the embryonic midbrain (A) The top 1,000 most variable genes of Seurat integrated object of merged scRNA-seq datasets of embryonic cortex (EC), embryonic midbrain (EM), midbrain organoids 35 days of differentiation (MO_WT35), and midbrain organoids 70 days of differentiation (MO_WT70). The average gene expression visualized after *Z* score normalization. (B) Expression of midbrain markers *FOXA2*, *TH*, *EN1*, and *EN2* in Seurat integrated object of merged scRNA-seq datasets of embryonic cortex (EC), embryonic midbrain (EM), midbrain organoids 35 days of differentiation (MO_WT35), and midbrain organoids 70 days of differentiation (MO_WT70). Each dot represents a single cell. (C) The average common gene expression correlation between midbrain organoids 35 days of differentiation (MO_WT35) and midbrain organoids 70 days of differentiation (MO_WT70) and embryonic midbrain (EM) developmental weeks (w6–w11). The Pearson correlation coefficient is displayed above each comparison. The highest correlation between midbrain organoids and embryonic developmental time point is highlighted in red. Each dot represents a single cell.

The transcriptome datasets of midbrain organoids, embryonic midbrain, and embryonic prefrontal cortex were embedded into a single Seurat object (Figure S3A). The average expression of the top 500 variable mutual genes showed a clear separation of the embryonic prefrontal cortex from midbrain organoids and the embryonic midbrain (Figure 1A). This separation indicates the expected greater similarity between midbrain organoids and the embryonic midbrain than the embryonic prefrontal cortex. The following correlation analysis of the average expression of all common genes confirmed that the transcriptome of midbrain organoids is more similar to the embryonic midbrain (ρ > 0.7) than to the embryonic prefrontal cortex (ρ < 0.7) (Figure S3B). Moreover, the embryonic midbrain and midbrain organoids express typical midbrain markers, such as *TH*, *FOXA2*, *EN1*, and *EN2*, which were absent or expressed at low levels in the embryonic prefrontal cortex (Figure 1B). The expression of TH, FOXA2, and EN1 in midbrain organoids was also validated by immunofluorescence staining (Figure S3C). In addition, we aimed to associate midbrain organoids to different time points in embryonic midbrain development by comparing the expression of the common genes between both datasets. The WT35 midbrain organoids highly correlated with embryonic week 9 (R = 0.92), while the midbrain organoids WT70 highly correlated with the week 10 (R = 0.90) (Figure 1C). These findings not only suggest that *in*-*vitro*-derived midbrain organoids show high gene expression similarities with the human embryonic midbrain but also manifest a developmental pattern comparable to their *in vivo* counterpart. In order to further validate the brain regional specificity of the organoids, we compared the scRNA-seq data of the embryonic midbrain to a cortex organoid^29^ in the same manner (Figure S3D). The Pearson correlation coefficient of 0.05 showed insignificant correlation between the embryonic midbrain and the cortex organoid, providing evidence that organoids derived from different brain regions exhibit no close transcriptome similarities with the developing embryonic midbrain *in vivo*.

### Midbrain organoids inherit physiological-relevant cellular populations that are shared with the developing embryonic midbrain

**Figure 2:**
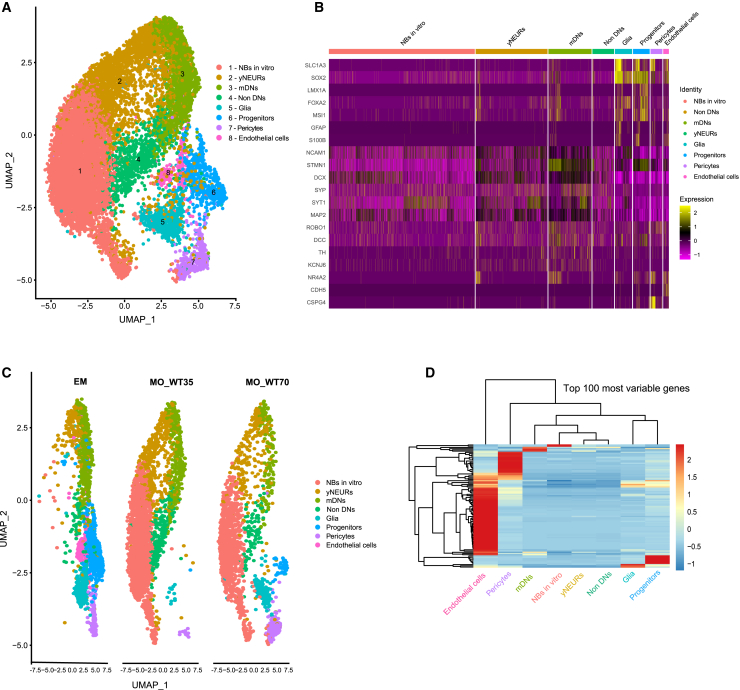
Midbrain organoids inherit physiological-relevant cellular populations that are shared with the developing embryonic midbrain (A) UMAP of integrated Seurat object of merged scRNA-seq datasets of embryonic midbrain, and WT and MUT midbrain organoids 35 and 70 days of differentiation, showing cell clusters 1–8, after manual correction of oversampling. Each dot represents a single cell and is colored according to the cell identity. (B) Identity heatmap showing cell-type-specific marker expression in identified cell clusters. (C) UMAP of cell clusters in embryonic midbrain (EM), WT midbrain organoids of 35 days of differentiation (MO_WT35), and 70 days of differentiation (MO_WT70). Each dot represents a single cell and is colored according to the cell identity. (D) Unsupervised hierarchical clustering of cell types, using the average expression of the top 100 most variable genes, visualized after *Z* score normalization.

After confirming that midbrain organoids present a gene expression signature comparable to embryonic midbrain *in vivo*, we used the integration workflow from Seurat^31^ to identify shared cellular populations across the *in vivo* and *in vitro* midbrain systems. We integrated the scRNA-seq data of the embryonic midbrain with healthy control and LRRK2-p.Gly2019Ser-mutant midbrain organoids of both differentiation time points 35 and 70 days (WT35, WT70, MUT35, and MUT70, respectively). We identified eight different cell types and visualized them by using UMAP (Figure 2A). To define cellular identities, we used the cell type marker gene list proposed by La Manno and colleagues^25^ and compared it to the marker gene list per cluster of the integrated object (Figure S4). We verified each marker expression in every cell cluster identified in the integrated Seurat object. The number of marker genes that were present in the cell populations (corresponding to the cell types defined in La Manno et al., 2016^25^) are shown in Figure S5A. La Manno and colleagues^25^ reported the presence of 25 cellular identities in the embryonic midbrain, including several sub-clusters of radial glia, progenitors, and dopaminergic neurons. To simplify cell identification, we grouped all 25 cell identities in more generic cell type clusters, such as neurons (NEURs), neuroblasts (NBs), progenitors, glia, pericytes, and endothelial cells. Neurons were further separated in non-dopaminergic neurons (non-DNs) and dopaminergic neurons (DNs). Cell identities were assigned to cell populations within the integrated Seurat object on the basis of the highest number of marker genes defining each generic cell type (Figure S5A). Once these clusters were broadly defined, using the embryonic midbrain data,^25^ we verified and refined the assigned cell identities on the basis of additional cell type and maturity-specific marker expression (Figure 2B, Figure S5B). We confirmed the particularly high expression of neuronal maturity markers^34^ and dopaminergic markers^9^ in DNs. Therefore, we defined DNs as mature DNs (mDNs). The neuronal cluster presenting lower expression of maturity and neuronal-type-specific marker expression, we defined as young neurons (yNEURs) (Figure S5B). The vast majority of cells in the yNEUR cluster showed a stable expression of young neuronal markers such as *NCAM1*, *STMN1*, and *DCX* (Figure 2B). The mature neuronal marker *MAP2* as well as synaptic genes such as *SYP* and *SYT1* were expressed in the mDN and non-DN clusters. Lastly, expression of the DN markers *TH*, *KCNJ6*, and *NR4A2* as well as of the DN-specific synaptic markers *ROBO1* and *DCC* were confirmed in yNEURs and mDNs. Importantly, midbrain identity markers *FOXA2* and *LMX1A* were expressed in most of the cell types (glia, progenitors, yNEURs, and mDNs). The radial glia marker *SLC1A3* and neural progenitor markers *SOX2* and *MSI1* showed high expression in the glia and progenitor clusters, suggesting that glia cells are rather immature at this stage of embryonic midbrain development and, thus, display a genetic signature of early development in midbrain organoids. However, also more specific glial markers such as *GFAP* and *S100B* were already detectable in some of the cells. Endothelial cell identity was confirmed by the positive expression of the *CDH5*, while pericyte cells showed robust expression of the blood vessel development regulator *CSPG4*. Cells belonging to the NB cluster were positive for neural stem cell marker (*SOX2*) as well as immature (*DCX*) and mature neural *(SYT1*) and DN markers (*TH* and *KCNJ6*). However, none of these markers showed a constantly high expression among all cells in the NB cluster. This suggests that the identity of NBs is rather yet undefined and might be a specific feature of *in vitro* cultures, with the potential to develop into more mature neural cell types over time.

Further, we visualized UMAP embeddings of cell types and split them by datasets to reveal common and distinct cell types across embryonic midbrain and midbrain organoids (Figure 2C). Clusters of progenitors, yNEURs, mDNs, non-DNs, and glia were present in the embryonic midbrain as well as midbrain organoids, demonstrating that most cell types are common between the *in vitro* and *in vivo* midbrain systems. We observed that the NB cluster was present mainly in midbrain organoids and not in the embryonic midbrain and therefore was called NBs *in vitro*. Pericytes were found in midbrain organoids and the embryonic midbrain, however more mature endothelial cells were only present in the embryonic midbrain.

Next, we investigated the most variable gene expression pattern between the defined cell types (Figure 2D). The top 100 most variable genes led to a clustering of yNEURS, mDNs, non-DNs, and NBs together, confirming the common neuronal expression profile of these cell types. Pericytes and endothelial cells showed rather distinct genetic signature, consistent with the fact that these cells have non-neuronal identity. Glia and progenitors formed another separate cluster with a similar transcriptomic profile, implying again an early developmental stage of the glial cells.

### Differential gene expression analysis reveals a *LRRK2*-related PD phenotype in the mutant midbrain organoids

**Figure 3:**
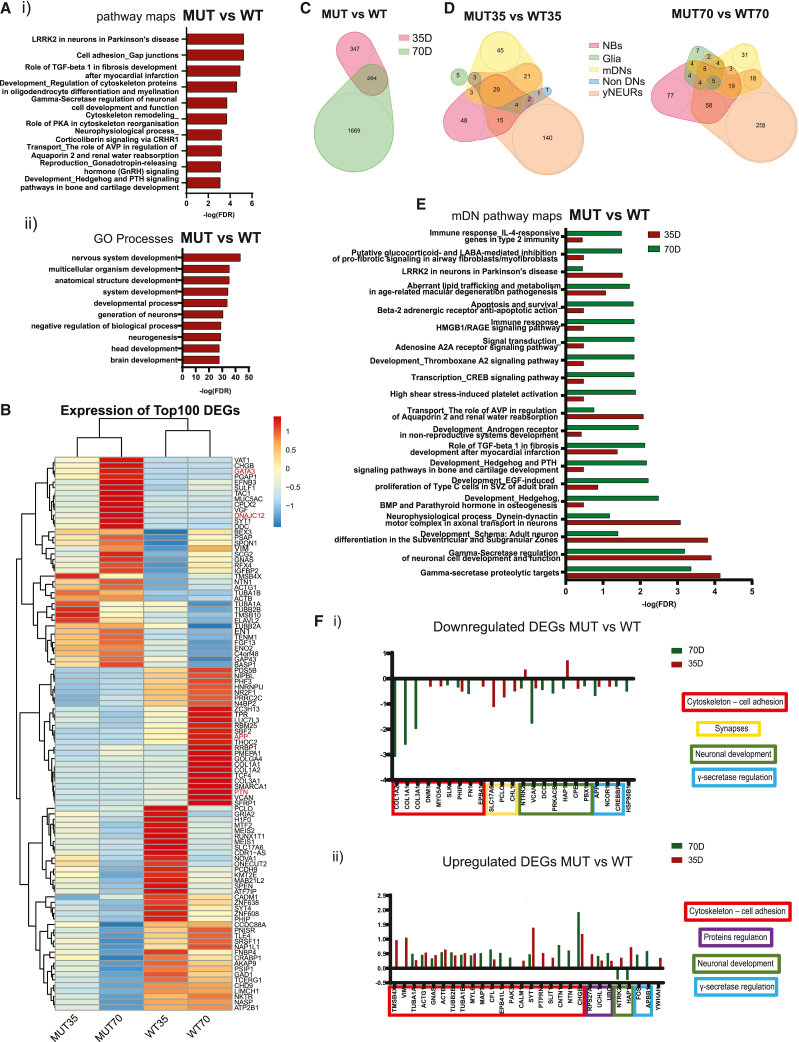
LRRK2-p.Gly2019Ser-mutant midbrain organoids recapitulate PD-associated pathways (A) Pathway maps (i) and GO processes (ii) of the enrichment analysis of 294 DEG (p adj. value < 0.05) between MUT and WT midbrain organoids. (B) Heatmap of to 100 DEG (p adj. value < 0.05) between MUT and WT midbrain organoids. Genes highlighted in red are the potential LRRK2 p.Gly2019Ser target genes involved in the neurodevelopment (see also Figure 6) (C) Venn diagram, showing the number of DEGs between MUT and WT midbrain organoids found at 35 days and 70 days of differentiation (p adj. value < 0.05). (D) Venn diagrams, showing the number of DEGs found in each cell type between MUT and WT midbrain organoids fat 35 days and 70 days (p adj. value < 0.05). (E) Mature DN pathway processes enrichment based on the DEGs identified in mDNs between MUT and WT midbrain organoids (p adj. value < 0.05). (F) Fold changes of genes selected from the top enriched pathways dysregulated in mDNs.

Further, we assessed the potential of midbrain organoids in disease modeling by comparing the transcriptomic signature of the midbrain organoids derived from the healthy control where the LRRK2 p.Gly2019Ser variant was inserted with the isogenic WT counterpart.^27^ As with the WT midbrain scRNA-seq data, we analyzed MUT midbrain organoid scRNA-seq data from organoids sampled at day 35 and day 70 of differentiation. We verified *LRRK2* expression in midbrain organoids and observed that it is expressed in a larger proportion of cells at later time points in both WT and MUT midbrain organoids (Figure S6A). In order to identify the key differences in the transcriptomic signature between MUT midbrain organoids and WT midbrain organoids, we computed the DEGs across both time points and all cell types with subsequent pathway enrichment analysis. The combined enrichment analysis of DEGs of both time points showed the most significant enrichment in the pathway of LRRK2 role in neurons in PD (Figure 3Ai). Moreover, other pathways associated with *LRRK2*, such as cytoskeleton regulation and cell adhesion, were also enriched in the MUT midbrain organoids. In addition, we found a significant DEG enrichment in protein kinase cAMP-dependent signaling and the γ-secretase regulation pathway. We identified that the most significant Gene Ontology (GO) and network processes were related to the neuronal development and axonal guidance (Figure 3Aii, Figure S6Bi). Furthermore, the most enriched diseases were linked to the brain and nervous system, confirming a diseased state of MUT midbrain organoids (Figure S6Bii). Last, the top 100 DEGs (adj. p value < 0.05) clustered MUT midbrain organoids separately from the WT midbrain organoids for both time points, confirming that LRRK2 p.Gly2019Ser induced changes in gene expression (Figure 3B). Interestingly, in the WT midbrain organoids, the expression levels of the DEGs differ between the two time points of differentiation, while in the MUT35 and MUT70 midbrain organoids, DEGs showed very similar expression patterns, indicating a potential developmental impairment of MUT organoids. Similarly, the top 100 DEGs separated the majority of different cell types of the MUT midbrain organoids from the WT midbrain organoids for both time points (Figure S5C), indicating that the presence of the *LRRK2* variant is responsible for gene expression changes in all cell types in at least one of the time points. However, the pathway enrichment analysis combined for all cell types showed a higher significance in the enrichment of cytoskeleton remodeling, γ-secretase regulation, and LRRK2-related pathways for day 70, suggesting a stronger manifestation of the LRRK2-p.Gly2019Ser-associated changes overtime (Figure S6D). In support of that, we identified in total 347 DEGs (adj. p value < 0.05) at day 35 and 1,669 DEGs (adj. p value < 0.05) at day 70 between the MUT and WT midbrain organoids. 264 DEGs were common between both time points (Figure 3C). Next, we overlapped all DEGs (adj. p value < 0.05) between cell types and saw that the highest number of DEGs at both time points were present in NBs *in vitro*, yNEURs, and mDNs (Figure 3D, Figure S7A). Pathway enrichment analysis identified that the cytoskeleton-regulation-related pathways were significant in the MUT35 and MUT70 midbrain organoids in all three respective cell types, while LRRK2-PD-related pathway occurred to be highly significant in NBs *in vitro*. (Figure 3E, Figure S7Bi). In mDNs and yNEURs, the γ-secretase and neurodevelopmental regulation pathways were identified as the most enriched for both time points (Figure 3E, Figure S6Bii), additionally indicating a possible link between LRRK2 p.Gly2019Ser and γ-secretase function.

In order to investigate the gene expression profiles between the MUT and WT midbrain organoids in more detail, we visualized the fold changes of the genes involved in the most significantly enriched pathways (Figures 3Fi and 3Fii). Genes related to cytoskeleton dysregulations, such as *COL1A2*, *COL1A1*, *COL3A1*, *DNM1*, *MYO5A*, *PHIP*, *SLK*, *FN1*, and *EPB41*, were found to be downregulated with a log2FC between −0.26 and −3, while others, such as *TMSB4X*, *VIM*, *TUBA1A*, *ACTG1*, *GNAS*, *TUBB2B*, *TUBA1B*, *MYL6*, *MAPT*, *CFL1*, *EPB41L1*, *PAK3*, *CALM1*, *SYT1*, *PTPRN*, *SLIT1*, *CNTN1*, *NTN1*, and *CHGB*, were found upregulated (log2FC between 0.26 and 1.93) in MUT midbrain organoids at the majority of both time points. Synapses-related genes, such as *SLC17A6*, *PCLO*, and *CHL1*, were particularly downregulated (log2FC between −0.5 and −1.12) in MUT35 midbrain organoids, but they were not differentially expressed in MUT70 midbrain organoids. Genes that are associated with neuronal development, such as *NTRK2*, *VCAN*, *DCC*, *PRKACB*, *HAP1*, *CPE*, and *PBX1*, were also dysregulated in MUT midbrain organoids. The majority of them were downregulated (log2FC between −0.38 and −1.77) in MUT70 midbrain organoids, while *NTRK2* and *HAP1* were upregulated (log2FC 0.36 and 0.72, respectively) in MUT35 midbrain organoids. Protein regulation-associated genes, such as *RPS27A*, *UCHL1*, and *UBC*, were upregulated (log2FC between 0.25 and 0.52) at both time points. Additionally, genes that are related to the γ-secretase regulation pathway, such as *APP*, *NCOR1*, and *CREBBP*, were downregulated (log2FC between −0.31 and −0.68) in MUT35 and MUT70 midbrain organoids, but *FOS* and *APBB1* were upregulated (log2FC 0.47 and 0.59, respectively), particularly in MUT70 midbrain organoids. We also observed a dysregulation of *HSP90B1*, which was downregulated (log2FC −0.51) at MUT70 midbrain organoids and *YWHAH* showing upregulation (log2FC 0.36) in MUT35 midbrain organoids. These genes encode HSP90B1 and 14-3-3 family proteins, respectively, known as direct interacting partners with LRRK2.

### Mutant midbrain organoids have a distinct cellular composition and correlate differently with the stages of embryonic development

**Figure 4:**
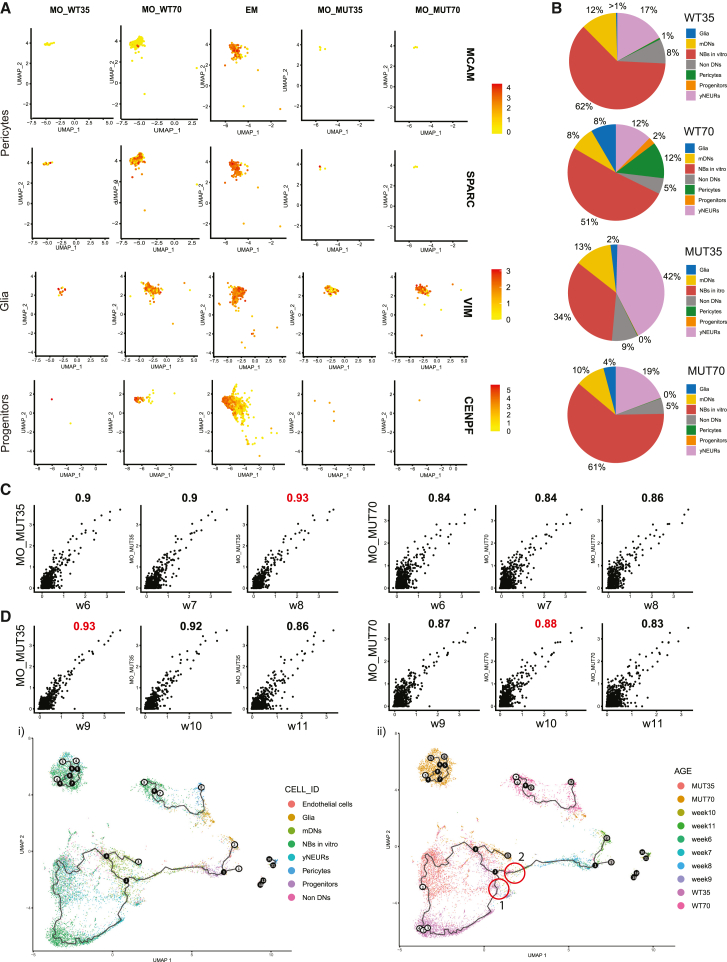
LRRK2-p.Gly2019Ser-mutant midbrain organoids have a different cellular composition and correlate differently with the stages of embryonic midbrain development (A) Cell cluster identities defined by typical marker expression between WT and MUT midbrain organoids for pericytes, glia, and progenitor cells. Each dot represents a single cell and is colored according to the expression level. (B) Percentage of cell identities in WT35, WT70, MUT35, and MUT70 midbrain organoids. (C) The average gene expression correlation between MUT midbrain organoids 35 days of differentiation (MO_MUT35) and 70 days of differentiation (MO_MUT70) compared to the embryonic midbrain (EM) developmental weeks (w6–w11). The Pearson correlation coefficient is displayed above each comparison. Each dot represents a single cell. (D) Batch-corrected pseudotime analysis based on the 150 dimensions. Each dot represents a single cell. The starting point is WT35 NBs *in vitro*. Cell distribution along the trajectory colored by cell identities (i) and by datasets (ii). Black nodes define branchpoints of the trajectory, white nodes define trajectory graph nodes, and gray nodes define endpoints of the certain trajectory leaf. Red circles indicate the position of mDNs of WT35 and WT70 midbrain organoids.

We observed that MUT midbrain organoids differ from WT midbrain organoids in their cellular composition. In the UMAP embedding plot split by models and colored by cell types (Figure S8A), we saw that progenitors and pericytes, which are shared cellular populations between WT midbrain organoids and embryonic midbrain, are not present in MUT midbrain organoids at any time point. On the contrary, we observed that the glia population is more enriched in MUT35 than in WT35 midbrain organoids. To confirm our observations, we subset the integrated Seurat object by the respective cell clusters and plotted them separately in the embryonic midbrain and in the WT as well as in the MUT midbrain organoids for both time points (Figure 4A). We saw that pericytes positive for the endothelial lineage marker *MCAM* and for the major regulator of angiogenic events, *SPARC*, are highly represented in WT70 midbrain organoids and in the embryonic midbrain but not in MUT midbrain organoids. Similarly, progenitors positive for the G2-proliferation-associated *CENPF* marker were only detected in the embryonic midbrain and WT70 midbrain organoids. A higher number of glia cells expressing *VIM* were already detected in MUT35 midbrain organoids compared to WT35 midbrain organoids. However, an increase of glia over time is more evident in WT than in MUT midbrain organoids.

Next, we calculated the proportion of each cell type present in WT and MUT midbrain organoids at both time points (Figure 4B). We saw a reduction of NBs *in vitro* (62%→51%) and yNEURs (17%→12%) from WT35 to the WT70. This reduction of less mature cells in WT35 midbrain organoids resulted in an increased variety of cell types present in WT70 midbrain organoids. Moreover, the cellular profile of WT70 midbrain organoids was quite similar to the cellular diversity observed in embryonic midbrain (Figure S8B). The major difference here was a high percentage of progenitors in the embryonic midbrain that seemed to be replaced by the presence of NBs *in vitro* in WT70 midbrain organoids.

Contrary to the WT midbrain organoids, in the MUT midbrain organoids, there was no evident difference in cell-type evolution over time. The same cell types were present in the MUT35 and MUT70 midbrain organoids, besides the fact that NBs *in vitro* almost doubled over time. Furthermore, the average gene expression correlation between MUT midbrain organoids and embryonic midbrain developmental time points showed that MUT35 midbrain organoids correlated better with embryonic development for all time points, compared to WT35 midbrain organoids (Figure 1C, Figure 4C). On the other hand, MUT70 midbrain organoids had a weaker correlation with the embryonic midbrain than the WT70 midbrain organoids, especially for week 11, which is also the latest and therefore most mature time point (R WT = 0.89 versus R MUT = 0.83). All together, these finding suggest that MUT midbrain organoids have a different developmental path compared to WT midbrain organoids and embryonic midbrain.

To further investigate the developmental differences between the MUT and WT midbrain organoids, we computed pseudotime trajectories to explore pseudotemporal ordering of midbrain organoid cell populations compared to the embryonic midbrain developmental time points. As the root, we chose WT35 NBs *in vitro* and we visualized the trajectories in UMAP plots colored by cell types and developmental time points of midbrain organoids and embryonic midbrain (Figures 4Di and 4Dii). We observed that mDNs of WT35 midbrain organoids are placed closer to embryonic developmental week 9 (branch point 2 and red circle 1 in Figure 4Dii), while mDNs of WT70 midbrain organoids were closer to embryonic developmental week 10 (branch point 2 and red circle 2 in Figure 4Dii), which is consistent with the gene average expression correlation analysis between WT midbrain organoids and embryonic midbrain. In clear contrast to this, we observed that mDNs of MUT35 and MUT70 midbrain organoids are placed closely to each other and formed a separate branch (branch point 3 to the endpoint 3 in Figure 4Di), which did not align with the embryonic midbrain trajectory. Further, we observed that glia cells of MUT70 and WT70 midbrain organoids (endpoint 2 and endpoint 8 in Figure 4Di) were arranged in close proximity to embryonic week 11 (endpoint 2 in Figure 4Dii), presenting appropriate developmental pattern, where gliogenesis follows neurogenesis. The similar distribution of MUT and WT glia within the pseudotemporal space indicates that the previously observed stagnation in glial development is linked to its number and not its quality. In general, the cells of MUT70 midbrain organoids were placed further from the embryonic developmental trajectory in the UMAP plot than the cells of WT70 midbrain organoids. This indicates that MUT midbrain organoids manifest a developmental deviation, while the development of WT midbrain organoids is more similar to embryonic midbrain *in vivo*. Moreover, MUT70 midbrain organoids demonstrated a more cyclic trajectory, confirming a limited cellular developmental path that is resulting in less variable cellular identities.

### Mutant midbrain organoids compared to wild-type midbrain organoids show impaired pseudotemporal development that manifests in an untimely and incomplete differentiation

**Figure 5:**
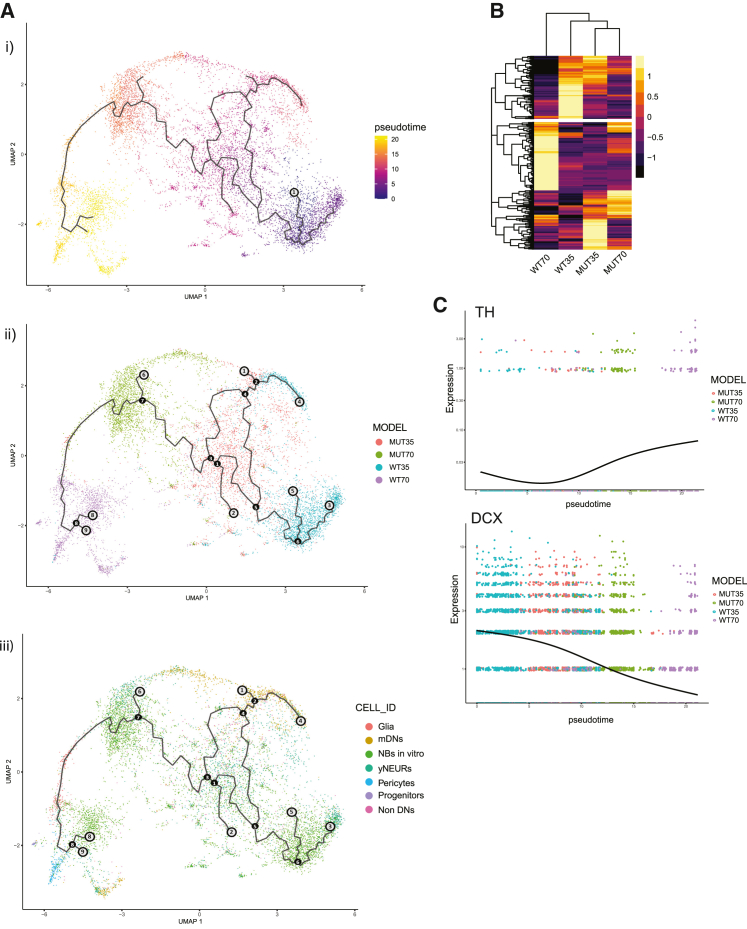
Transcriptome signatures over time reveal impaired development of LRRK2-p.Gly2019Ser-mutant midbrain organoids (A) Pseudotime analysis of midbrain organoids with the root node WT35 NBs *in vitro* (i). Pseudotime trajectory, cells colored by the model: WT35, WT70, MUT35, and MUT70 (ii). Pseudotime trajectory, cells colored by cell identity (iii). Black nodes define branchpoints of the trajectory and gray nodes define endpoints/outcomes of the certain trajectory leaf. (B) Genes with fitted expression pattern along the trajectory between WT35 and WT70 midbrain organoids, visualized in heatmap after *Z* score normalization in WT and MUT organoids. (C) Pseudotemporal expression of *TH* and *DCX* across the cells in WT and MUT midbrain organoids. Each dot represents a single cell.

In order to further explore the developmental deviation of the MUT midbrain organoids from WT midbrain organoids, we computed a developmental pseudotime trajectory only across midbrain organoids (WT35, WT70, MUT35, and MUT70), excluding the embryonic midbrain. The cell distribution along the trajectory starting from NBs *in vitro* of WT35, demonstrated accelerated differentiation of MUT35 midbrain organoids with subsequent developmental withhold (Figure 5A). We observed that mDNs might be the most affected cellular population. We saw that mDNs of WT midbrain organoids follow a differentiation path along the pseudotime trajectory from WT35 to the edge of WT70 midbrain organoids (endpoint 4 to 9). Contrary, mDNs of MUT35 midbrain organoids were located in close proximity to mDNs of MUT70 midbrain organoids (between endpoints 1 and 6), implying the impaired mDN maturation. In addition, glia cells of MUT35 midbrain organoids were located close to the WT70 midbrain organoids on the pseudotime trajectory (between branch points 7 and 8), confirming forwarded glia differentiation of MUT35 midbrain organoids.

Next, we computed genes with a clear expression switch across the developmental trajectory between WT35 and WT70 midbrain organoids (Figure 5B, Figure S8C). We investigated whether the same genes that have a temporal dynamic expression pattern in WT midbrain organoids show similar expression tendency in MUT midbrain organoids (Figure 5B). We observed that MUT midbrain organoids presented a completely different expression of the same genes, suggesting that MUT midbrain organoids do not follow the same developmental process as WT midbrain organoids. Furthermore, we highlighted the temporal expression of the rate-limiting enzyme of dopamine synthesis, tyrosine hydroxylase (*TH*), and the developing neuronal marker doublecortin (*DCX*). *TH* expression showed an increase over time in WT midbrain organoids but was impaired in the MUT midbrain organoids at both time points. *DCX* showed a clear decrease in the expression between WT35 and WT70 midbrain organoids. While in MUT35 midbrain organoids its expression was already further declined, it was still expressed at MUT70 midbrain organoids, further supporting an accelerated differentiation in MUT midbrain organoids at early time points of development accompanied by an incomplete differentiation at later developmental stages. These results imply that mutant midbrain organoids reach a deadlock at some point during development.

### Identification of potential LRRK2 p.Gly2019Ser target genes that could underlie impaired neurodevelopment and contribute to explain the PD-associated genetic signature

**Figure 6:**
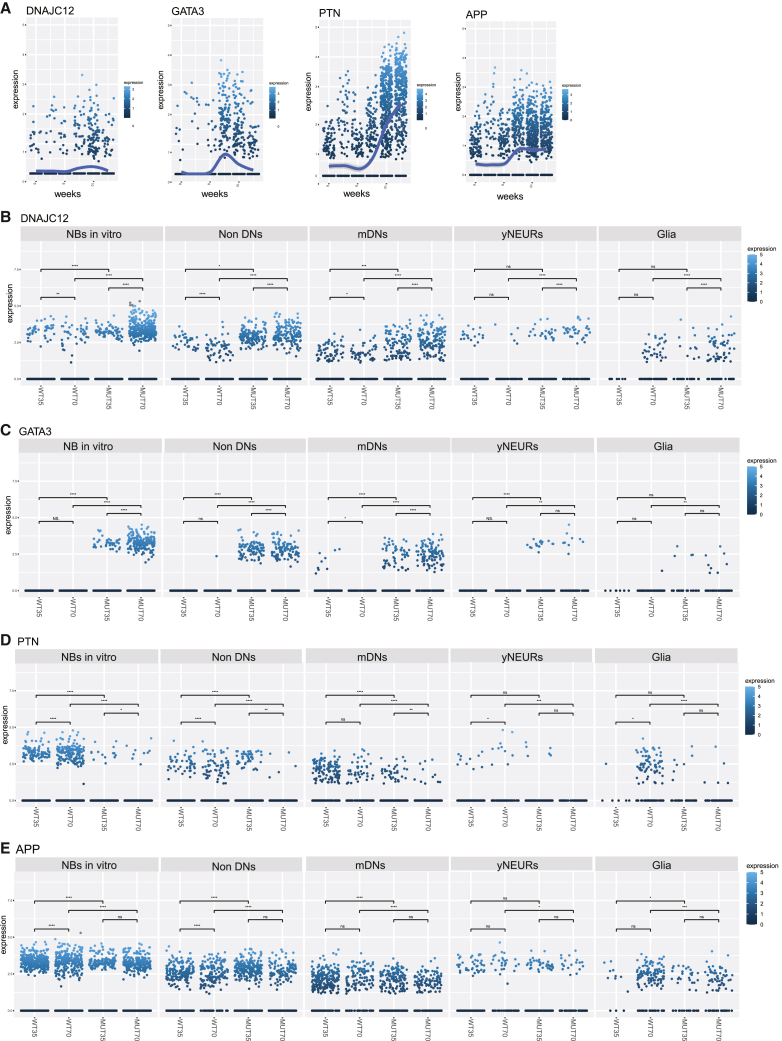
Discovery of potential LRRK2 p.Gly2019Ser target genes that might be involved in impaired neurodevelopment of mutant midbrain organoids (A) *DNAJC12*, *GATA3*, *PTN*, and *APP* expression profile over the embryonic development time points (w6–w11). Each dot represents a single cell of embryonic midbrain and is colored according to the expression level. (B–E) *DNAJC12*, *GATA3*, *PTN*, and *APP* expression across major cell types in WT and MUT midbrain organoids at 35 days and 70 days of differentiation. Each dot represents a single cell of midbrain organoid and is colored according to the expression level. Kruskal-Wallis test p < 0.05^∗^, p < 0.01^∗∗^, p < 0.001^∗∗∗^, p < 0.00001^∗∗∗^.

On the basis of the DEG analysis and after pseudotime trajectory examination, we distinguished four potentially promising candidate genes that have already been associated with PD^35^, ^36^, ^37^, ^38^, ^39^, ^40^—*DNAJC12*, *GATA3*, *PTN*, and *APP* (Figure 3B). These genes showed a temporal dynamic expression in the developing embryo and were significantly differentially expressed in MUT midbrain organoids compared to WT midbrain organoids. Their considerable change in expression during embryonic development indicates an active role in neurodevelopment (Figure 6A). Moreover, differential expression between MUT and WT midbrain organoids further supports altered MUT midbrain organoid neurodevelopment. *DNAJC12* and *GATA3* showed a significant upregulation in every neuronal cell type and glia in MUT midbrain organoids compared to WT midbrain organoids in both time points (Figures 6B and 6C). In addition, in MUT organoids, *DNAJC12* and *GATA3* expression increased over time in contrast to the embryonic midbrain where the expression decreased after peaking at week 9. In the neuronal clusters of WT midbrain organoids, the expression pattern of these two genes was comparable to embryonic development, showing highest expression levels at 35 days (corresponding to week 9). In contrast, *PTN* and *APP* were found to be significantly downregulated in MUT midbrain organoid neuronal cell types and glia compared to WT midbrain organoids (Figures 6D and 6E). We observed that both *PTN* and *APP* expression tended to increase over time in embryonic midbrain development. A similar expression pattern was observed in NBs *in vitro*, yNEURs, and glia of WT midbrain organoid but not in MUT midbrain organoids. These results highlight a dysregulation of genes with essential roles in neuronal development and neuroprotection that might be directly associated with the LRRK2 p.Gly2019Ser variant, linking *LRRK2* to the regulation of nigrostriatal system development.

## Discussion

Our analysis of scRNA-seq data of human midbrain organoids and embryonic midbrain highlights the physiological relevance of midbrain organoids and their potential in disease modeling. Over the recent years, midbrain organoids have become a widely used model in PD studies, as the midbrain is the most affected region in the brain of PD patients.^9^^,^^20^^,^^23^^,^^41^^,^^42^ In the present study, we were able to show the developmental correlation of healthy control-derived midbrain organoids from 35 and 70 days of culture^9^ with human embryonic midbrain.^25^ Importantly, midbrain organoids showed a higher degree of correlation with embryonic midbrain development than with the embryonic prefrontal cortex, validating the midbrain identity of the organoids. In support of this, we did not find a significant correlation between the cortex organoids^29^ and embryonic midbrain, which further validates the specificity of the brain regional organoids. In addition, our analysis implicates developmental maturation of midbrain organoids after long time culture (e.g., 70 days), which showed a better correlation with the later stages of embryonic midbrain development.

Previous studies have demonstrated the cellular heterogeneity of human brain organoids and their similarities with their fetal counterparts.^43^^,^^44^ Similarly, our analysis showed that midbrain organoids exhibit a shared cellular composition with the developing embryonic midbrain. One interesting finding is the identification of pericytes in midbrain organoids. It has been reported that pericytes can originate from the neuroectoderm and contribute to the formation of vasculature in the CNS.^45^, ^46^, ^47^ The presence of mesenchymal cells was also originally reported by Smits et al., 2020.^26^ Moreover, recent studies showed that a mesenchymal-like cell population appears in the early development of the cortex, even before the beginning of neurogenesis.^48^ Nevertheless, because the cells in midbrain organoids are guided toward midbrain identity by the expansion of the neuroepithelium, the presence of more mature endothelial cells is not expected. Accordingly, the endothelial cell cluster was found only in the embryonic midbrain and not in midbrain organoids. In contrast, the NB *in vitro* cluster was almost uniquely present in the midbrain organoids. Although these cells did not show a significant variable gene expression profile and clustered with neuronal cell types, there was no expression of reliable marker genes. Due to their unclear gene expression profile, NBs *in vitro* seemed to be less comparable to the physiological cell types shared between midbrain organoids and embryonic midbrain. We speculate that this NB cluster represents mfNPCs, which is the starting cell population for midbrain organoid generation. Although these cells are artificially patterned toward midbrain identity^49^ and show unspecific genetic identity,^50^ they can give rise to multiple physiologically relevant neuronal cell types and glia, similar to their *in vivo* neural progenitor counterpart.

When comparing MUT to WT midbrain organoids, clear differences become visible regarding their cellular composition, revealing PD-associated phenotypic differences. The MUT midbrain organoids reveal a faster differentiation profile that limits the development of a more variable and mature cellular composition. The accelerated differentiation phenotype at 35 days that we observed with pseudotime analysis has been described before in *LRRK2*-related PD.^8^, ^9^, ^10^ In addition, the MUT midbrain organoids have no evident differences in the cell type populations at both time points and pseudotime analysis revealed that besides the untimely differentiation, the MUT70 midbrain organoids face a premature arrest or slowdown of the differentiation capacity. Importantly, the mDNs were the most affected population of cells. They showed no indication of maturation along the trajectory in the MUT70 compared to the MUT35 midbrain organoids and had a reduced expression of TH in MUT midbrain organoids. The doubling of the number of NBs *in vitro* in MUT70 midbrain organoids might be a compensation strategy linked to the incapacity of terminal differentiation or an increase in mature cell death. Moreover, we observed that the MUT midbrain organoids contain a higher number of glial cells than WT midbrain organoids at early time points. A situation that is inverted in longer cultures (MUT70 and WT70). The pseudotime trajectory confirmed that glial cells of MUT35 midbrain organoids were located closer to the WT70 midbrain organoids, indicating a faster gliogenesis. Finally, and most importantly, in contrast to MUT midbrain organoids, WT midbrain organoids from longer cultures are capable of capturing the cellular diversity found in human embryonic midbrain development *in vivo*.

Regarding the developmental pattern of organoids and embryonal tissue, MUT midbrain organoids showed a different developmental path compared to WT midbrain organoids. From the correlation analysis, we saw that the MUT70 midbrain organoids have lower correlation than MUT35 midbrain organoids with the different time points of embryonic midbrain development. Furthermore, cells of MUT70 midbrain organoids were positioned further away from the embryonic pseudotemporal developmental trajectory in the UMAP plot, while cells of WT70 midbrain organoids have a development trail closer to embryonic development.

On the basis of the here-presented data, we propose that LRRK2 p.Gly2019Ser could be responsible for the observed developmental defects and the impaired cellular composition. Our *LRRK2* midbrain organoid model was able to capture the dysregulation of gene expression linked to *LRRK2*-induced PD. The analysis of DEGs between MUT and WT midbrain organoids showed the significance of LRRK2-related pathway in PD and highlighted GO processes related to nervous system development. In addition to individual gene dysregulation of LRRK2-associated pathways, the overall DEG analysis showed a clear separation of the MUT and WT midbrain organoid clusters, confirming the presence of disease-associated phenotypes.

The major dysregulated pathways were cytoskeleton remodeling and cell adhesion. It is well known that LRRK2 plays an important role in actin and microtubule dynamics. LRRK2 p.Gly2019Ser has been reported to disturb the cytoskeleton processes through increased kinase activity.^51^^,^^52^ The dysregulation of actin and microtubule genes, which are key components of cytoskeleton dynamics, may lead to failure of the proper cellular differentiation process.^26^ Cytoskeleton-related proteins, such as MYO5A, DNM1, EPB41, ACTB, MAPT, and VIM, are direct interacting partners of LRRK2.^53^ We found that the corresponding genes have a dysregulated expression in the MUT midbrain organoids, indicating that altered LRRK2 function is able to impair the gene expression profile of its interactome. Altered LRRK2 function has also been described to have a role in impaired synaptogenesis.^51^^,^^54^^,^^55^ Here, we identified significant downregulation of the synapse-related genes *SLC17A6*, *PCLO*, and *CHL1* specifically in MUT35 but not in MUT70 midbrain organoids. This observation suggests an impaired synaptogenesis occurring in early neurodevelopment of MUT midbrain organoids.

Further, direct LRRK2-interacting partners such as HSP90B1 and YWHAH have also been altered upon presence of the LRRK2 p.Gly2019Ser. HSP90B1 along with the other heat-shock proteins is involved in protein folding and has been linked to PD.^56^ HSP90B1 is a chaperone protein from the HSP90 family that interacts with LRRK2. This interaction is important for the proteasomal degradation of LRRK2.^57^ Thus, the downregulation of *HSP90B1* in MUT70 midbrain organoids could be linked to the toxic aggregation of mutant LRRK2. *YWHAH* encodes the 14-3-3 eta, known to regulate the activity of kinases, including LRRK2.^58^

Additionally, we identified dysregulation of genes related to the γ-secretase pathway. APP belongs to this pathway and shows a severe dysregulation in MUT midbrain organoid. *APP* encodes the β-amyloid precursor protein that has an important role in the development of neurodegenerative pathologies such as Alzheimer disease because of the accumulation of its derivative amyloid-beta (Aβ) peptide, which is induced by cleavage from secretases including the γ-secretase.^59^^,^^60^ A link between Aβ accumulation and LRRK2 p.Gly2019Ser PD cases has also been made. LRRK2 phosphorylates the intracellular domain (AICD) of APP, which regulates the transcription of cytoskeleton-related genes and has a role in the loss of dopaminergic neurons in the midbrain of PD cases by induced neurotoxicity.^61^ APP has also an important role in neurogenesis, gliogenesis, and neuroprotection in the developing brain.^35^^,^^60^^,^^62^ Therefore, dysregulation of *APP* can be associated not only with the neurodegeneration but also with the aberrations of neuronal development. Indeed, during embryonic midbrain development, we observed a strong increase of *APP* expression over time, whereas in MUT midbrain organoids, we observed a significant reduction compared to WT midbrain organoids, especially in mDNs and glia cells.

Further evidence of altered cellular development of MUT midbrain organoids comes also from the changed expression of three PD-associated DEG candidates, *DNAJC12*, *GATA3*, and *PTN*. The expression pattern of these genes in the embryonic midbrain suggests their important role in development and differentiation of the cells, although these findings would benefit from further experimental validation. DNAJC12 is described to have a role in protein folding and export. Bi-allelic mutations of *DNAJC12* have been associated with hyperphenylalaninemia and neurodevelopmental delay in children. However, recent findings link mutation in *DNAJC12* to early-onset PD because of its interaction with aromatic amino-acid hydroxylases, including TH.^37^^,^^63^ GATA3 has been described as an important regulator of CNS development and neuronal fate.^36^ An association with PD has been reported via GATA family transcriptional regulation of *TRPM2* and *SNCA*.^38^^,^^64^ Until now, there is no reported interaction of GATA3 and DNAJC12 with LRRK2. However, the notable upregulation of *GATA3* and *DNAJC12* in MUT midbrain organoids suggests their possible dysregulation due to LRRK2 p.Gly2019Ser and might explain the accelerated differentiation phenotype, subsequent maturation decline, and decreased expression of *TH*. In contrast, we observed that *PTN* is expressed significantly higher in WT midbrain organoids. PTN is a neurotrophic factor, highly expressed during development of nigrostriatal dopamine system, and later plays a role in cellular recovery and repair.^39^^,^^40^ It has been shown to restore neuronal survival and functionality in a 6-OHDA mouse model.^39^ The high expression of *PTN* in NBs *in vitro* of WT midbrain organoids may explain their better developmental trail compared to MUT midbrain organoids.

In summary, we demonstrated a high degree of transcriptome similarity between human midbrain organoids and embryonic midbrain, supporting the potential of midbrain organoids to recapitulate human brain physiology. Moreover, our study showed the ability of midbrain organoids to capture LRRK2-p.Gly2019Ser-dependent alterations in gene expression, which highlights cellular processes related to cytoskeleton regulation, cell adhesion, and γ-secretase regulation during neuronal development. Finally, we observed developmental aberrations in MUT midbrain organoids and altered gene expression patterns along pseudotemporal trajectories, supporting a neurodevelopmental component in LRRK2-p.Gly2019Ser-associated PD.

## Data and code availability

WT midbrain organoid scRNA-seq datasets are available at the following doi: https://doi.org/10.17881/lcsb.20190326.01. LRRK2-G2019S MUT midbrain organoid scRNA-seq datasets are available at the following doi: https://doi.org/10.17881/rc4f-nk07. The accession number for the raw data for WT and MUT midbrain organoids is GEO: GSE133894. The accession number for the raw data for the human embryo ventral midbrain between 6 and 11 weeks is GEO: GSE76381. The accession number for the raw data for scRNA-seq data of prefrontal cortex at developmental stages between gestational weeks 8 and 26 is GEO: GSE104276. The accession number for the raw data for scRNA-seq data of human cortical organoids is GEO: GSE130238. We used data only of 1-month-old cortical organoid, which better corresponds to the developmental stage of midbrain organoids. Data were analyzed with R version 3.6.2 with single-cell analysis toolkit Seurat version 3.2.0^31^^,^^65^^,^^66^ and Monocle 3.^67^ All scripts used for the analysis are available via GitHub: https://github.com/LCSB-DVB.

## Web resources

Metacore, https://portal.genego.com/
nVenn, http://degradome.uniovi.es/cgi-bin/nVenn/nvenn.cgi

## Supplemental information

Document S1. Figures S1–S8 and Table S1

Document S2. Article plus supplemental information
