# Supplementary material for: Midbrain organoids mimic early embryonic neurodevelopment and recapitulate LRRK2-p.Gly2019Ser-associated gene expression
Source: Am J Hum Genet. 2022 Jan 24;109(2):311–27. doi: 10.1016/j.ajhg.2021.12.009 (PMC8874228; doi:10.1016/j.ajhg.2021.12.009)
Supplement: Document S1. Figures S1–S8 and Table S1 [file mmc1.pdf]

The American Journal of Human Genetics, Volume *109*

Supplemental information

Midbrain organoids mimic early embryonic neurodevelopment and recapitulate LRRK2-p.Gly2019Ser-associated gene expression

Alise Zagare, Kyriaki Barmpa, Semra Smajic, Lisa M. Smits, Kamil Grzyb, Anne Grünewald, Alexander Skupin, Sarah L. Nickels, and Jens C. Schwamborn

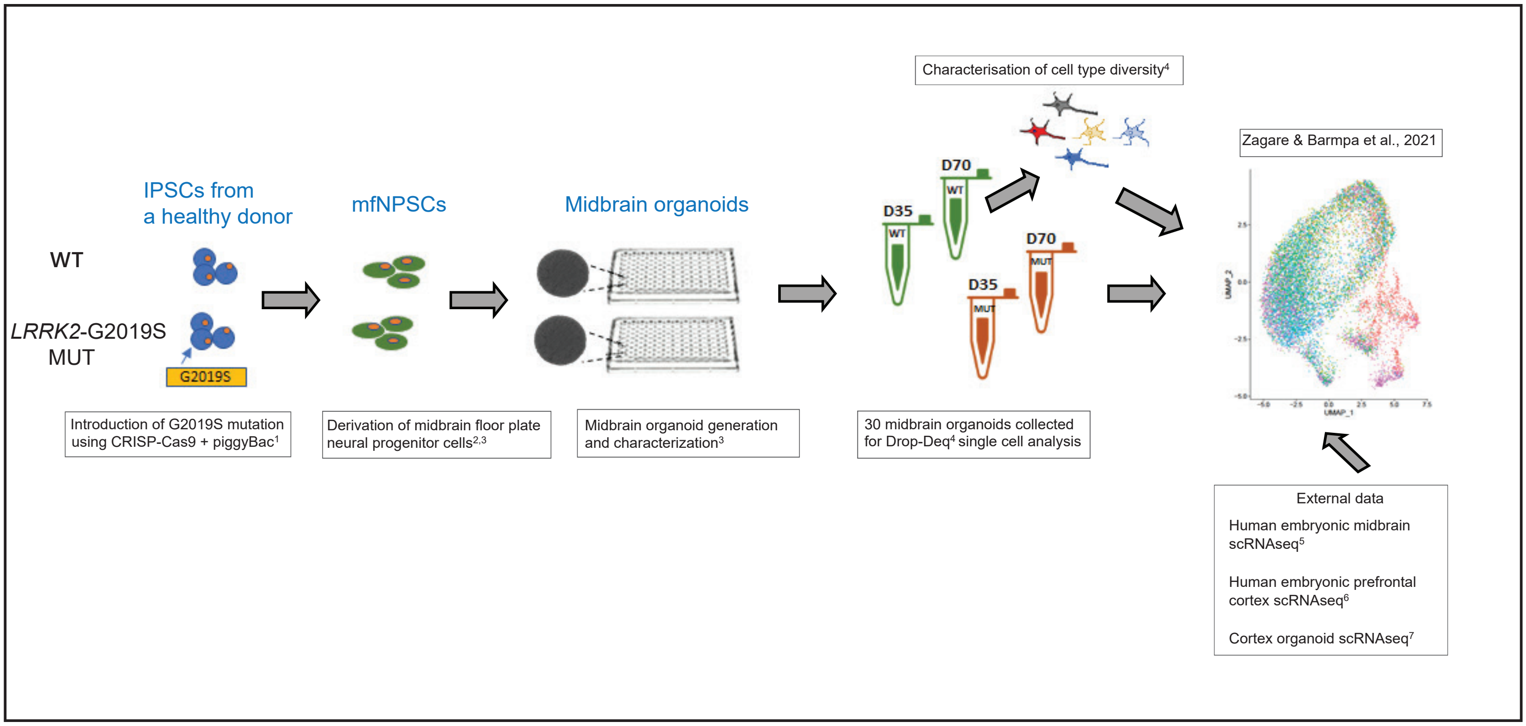

Figure S1. Graphical representation showing the origin of the data used in this study. WT and LRRK2 MUT midbrain organoids were generated in paralel in our lab. WT midbrain organoids were characterized in previous studies of Smits et al. [3,4] The present study comprises analysis of internal scRNAseq data from the respective midbrain organoids and comparison to external datasets [5,6,7].

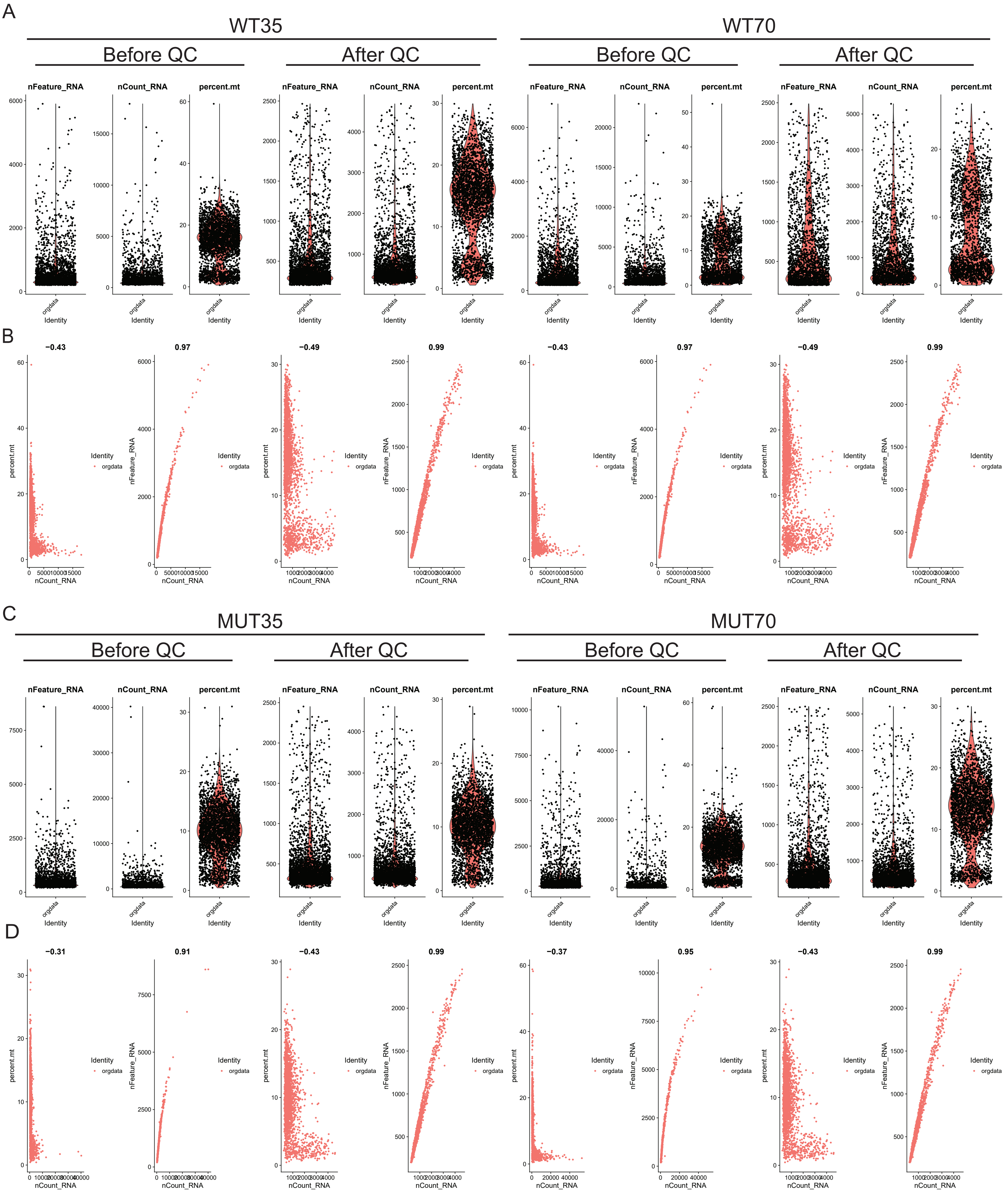

Figure S2. Quality control of midbrain organoid scRNAseq datasets. **A)** Number of genes (nFeature_RNA), total number of molecules (nCount), percent of mitochondrial genes, detected in each cell for WT35 and WT70 midbrain organoids, before and after quality control. **B)** Correlations between percentage of mitochondrial genes, the number of genes and the total number of molecules for WT35 and WT70 midbrain organoids, before and after quality control. **C)** Same as (A), for MUT35 and MUT70 midbrain organoids. **D)** Same as (B), for MUT35 and MUT70 midbrain organoids.

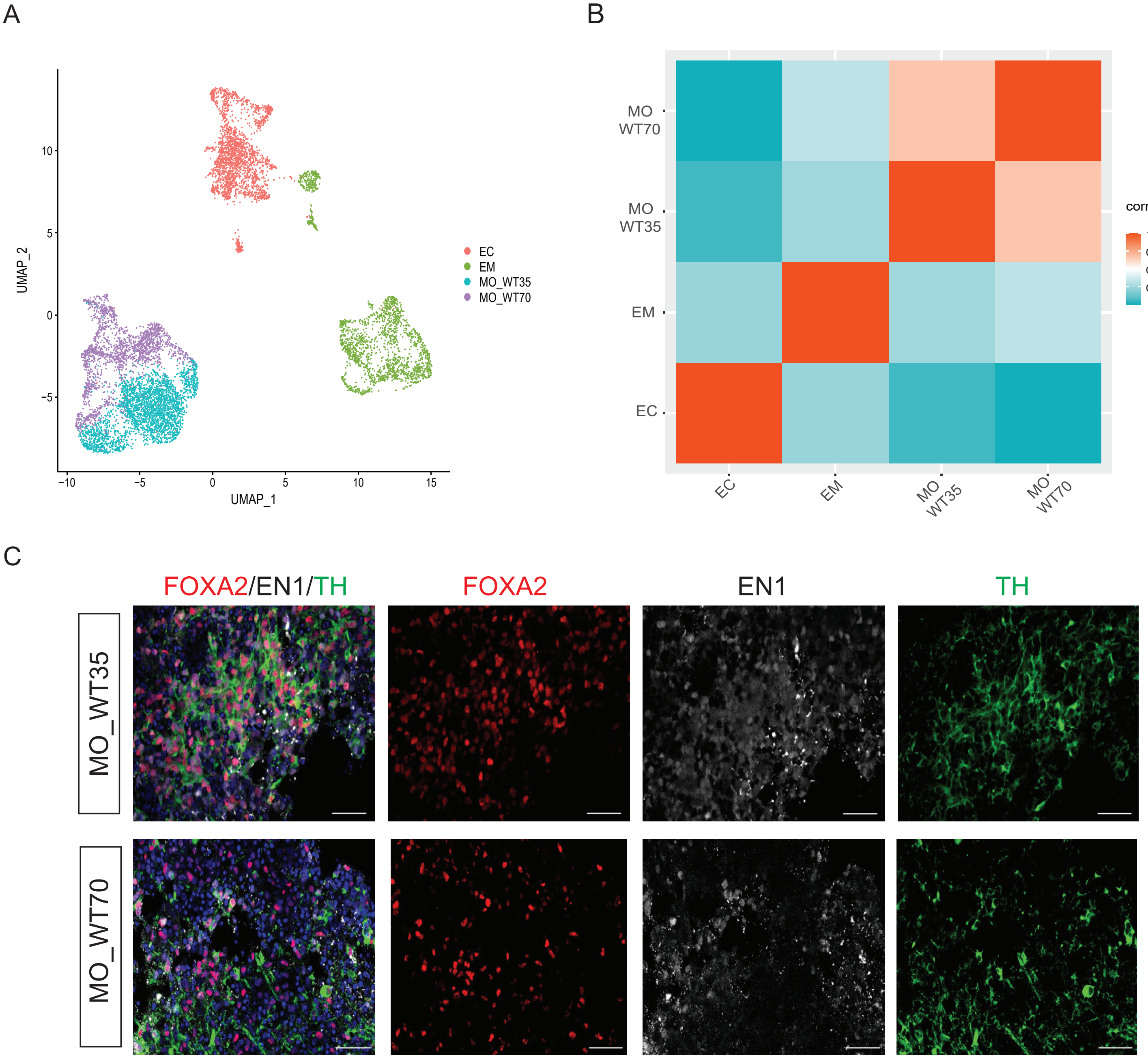

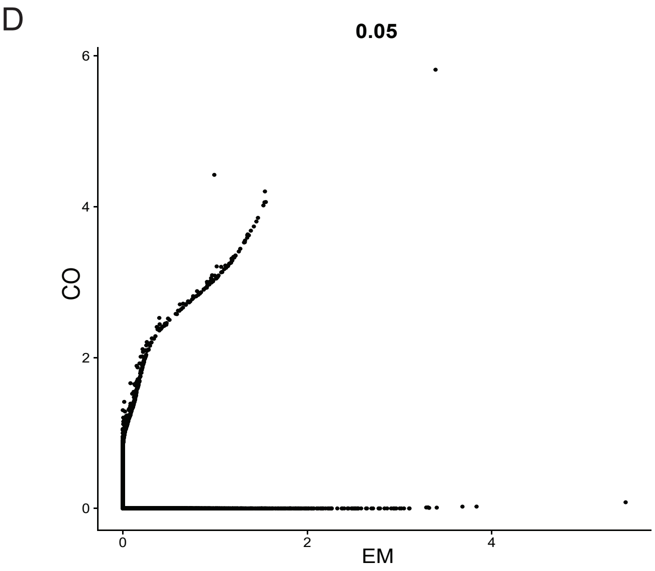

Figure S3. Validation of midbrain organoid identity. **A)** UMAP of integrated Seurat object of merged embryonic prefrontal cortex (EC), embryonic midbrain (EM), WT midbrain organoids of 35 days of differentiation (MO_WT35) and 70 days of differentiation (MO_WT70) after SCTransform. **B)** Spearman correlation of mutual gene average expression between the embryonic prefrontal cortex (EC), embryonic midbrain (EM), WT midbrain organoids of 35 days of differentiation (MO_WT35) and 70 days of differentiation (MO_WT70). **C)** Immunofluorescent staining of midbrain identity markers: FOXA2, EN1 and TH in midbrain organoid sections. Scale bar: 50µm. **D)** The average gene expression correlation of the non-integrated embryonic midbrain (EM) and cortical organoid (CO).

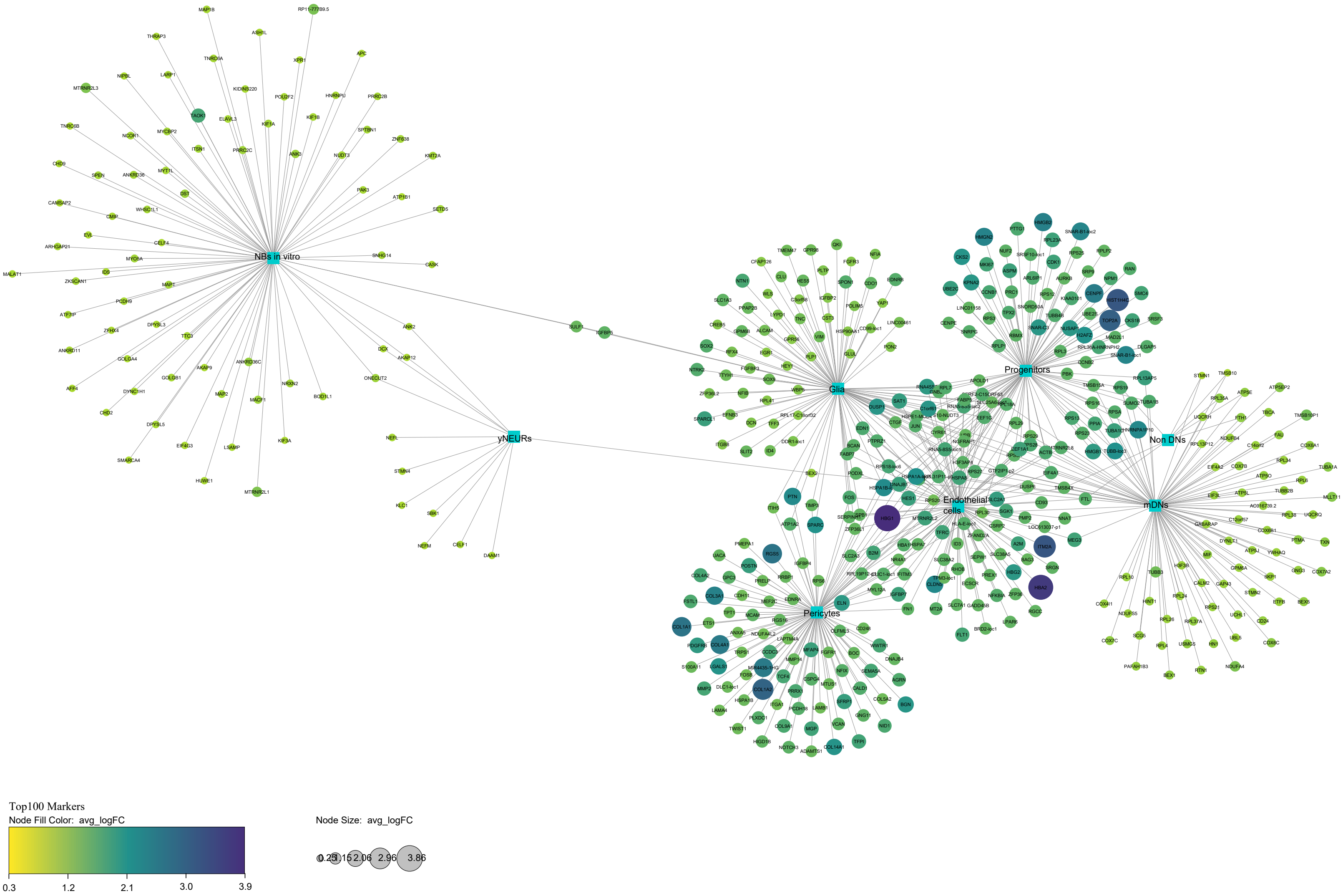

Figure S4. Network representing top marker genes for each cell cluster.
The top 100 cell type markers identified by the FindAllMarkers function. The size and color of nodes represent logarithmic fold change (avg_logFC) of each marker expression in the particular cell type compared to its expression in other cell clusters.

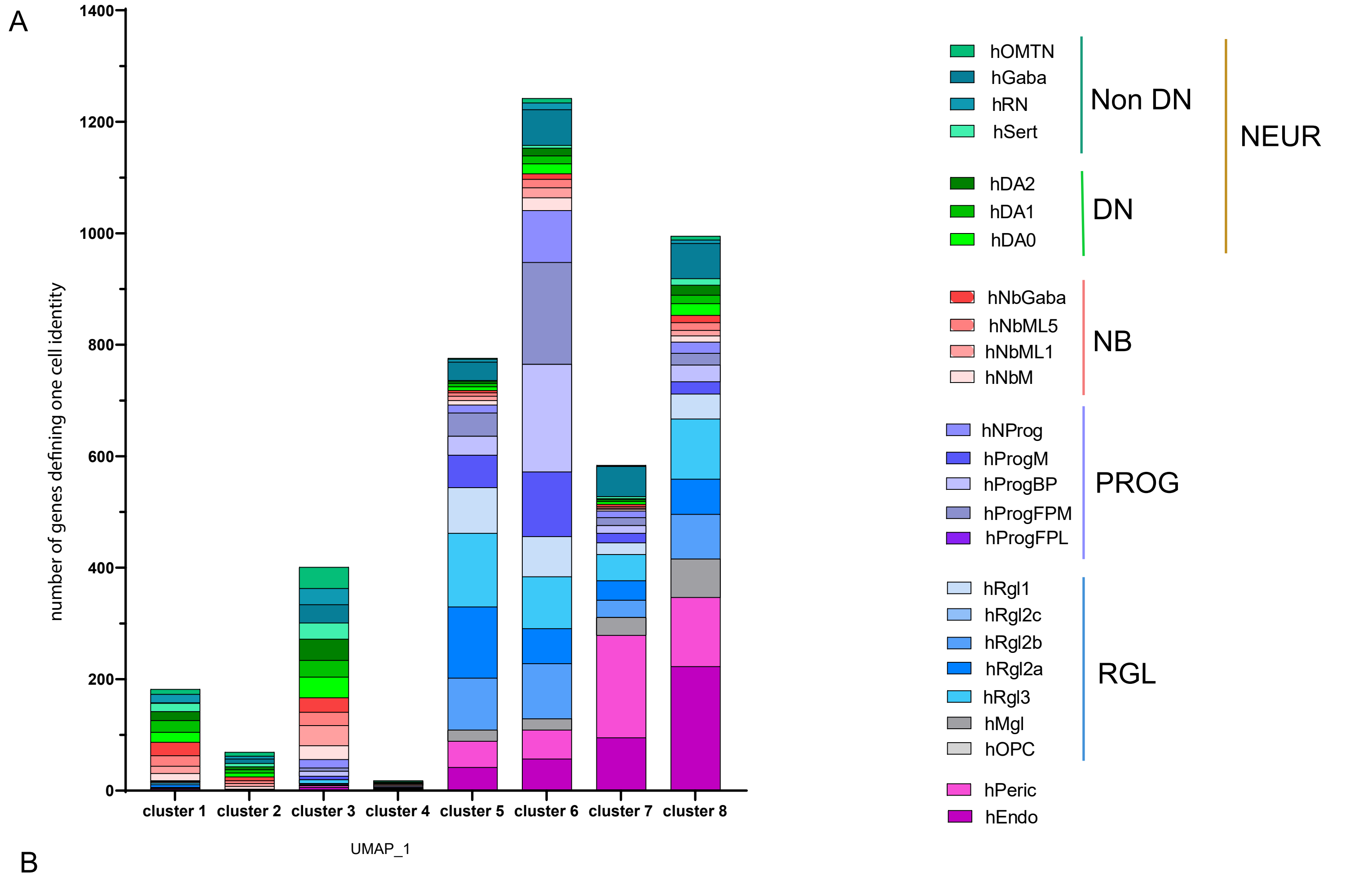

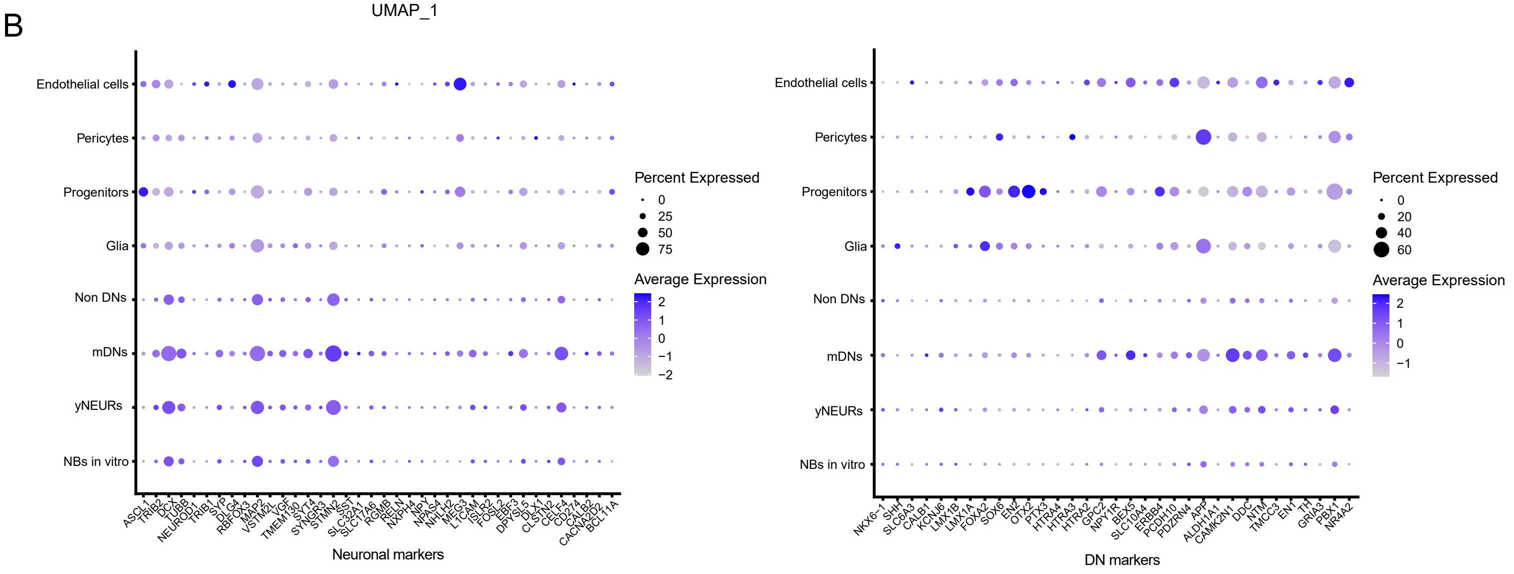

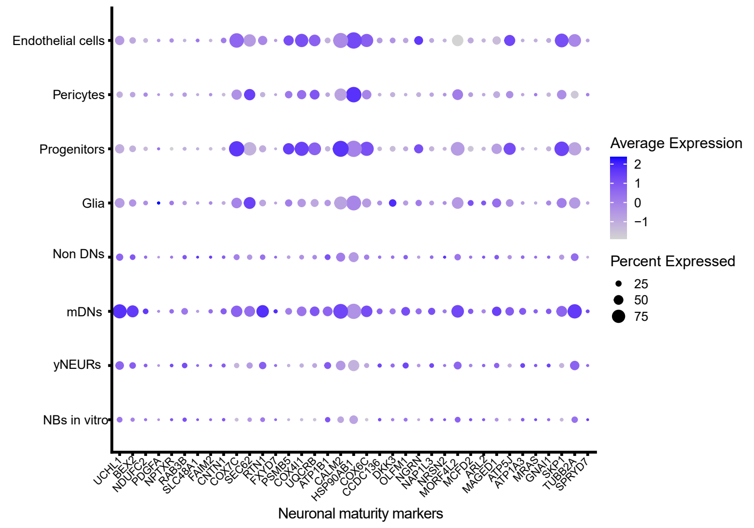

Figure S5. Cell cluster naming. **A)** Cell cluster annotation based on La Manno et al. 2016 [5] showing all embryonic cell names and the simplified regrouping. **B)** Neuronal marker, dopaminergic marker [8], and neuronal maturity marker [9] expression in defined cell types.

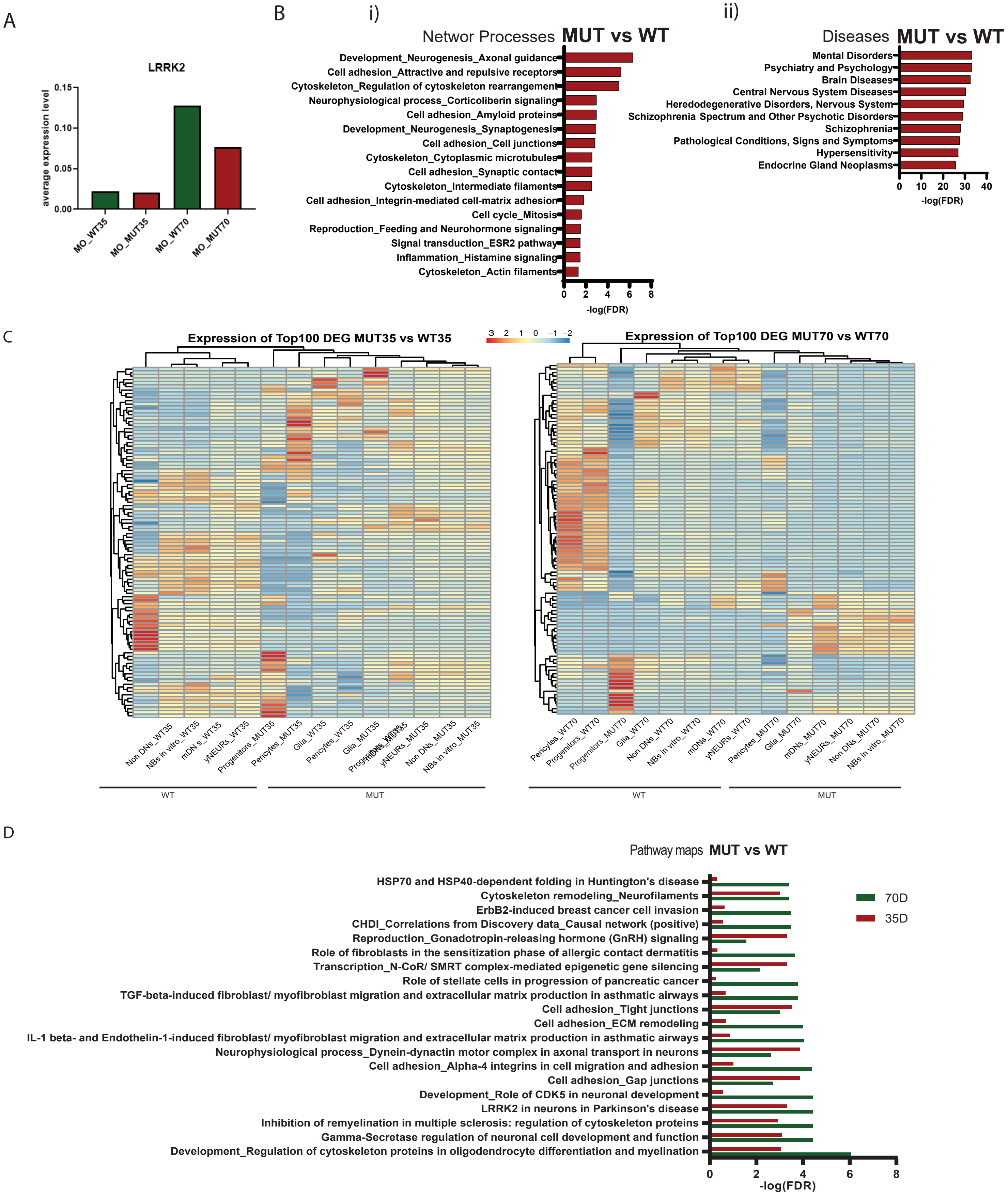

Figure S6. Differentially expressed gene analysis I. **A)** The bulk average *LRRK2* expression in midbrain organoids. **B)** i) Network processes and ii) diseases from the enrichment analysis of 294 DEGs (adjusted p-value < 0.05) between MUT and WT midbrain organoids. **C)** Heatmaps of top 100 DEGs (adjusted p-value < 0.05) at day 35 and day 70 respectively, showing the cell type unsupervised clustering between MUT and WT organoids. **D)** Pathway processes based on MUT vs WT midbrain organoid DEGs (adjusted p-value < 0.05).

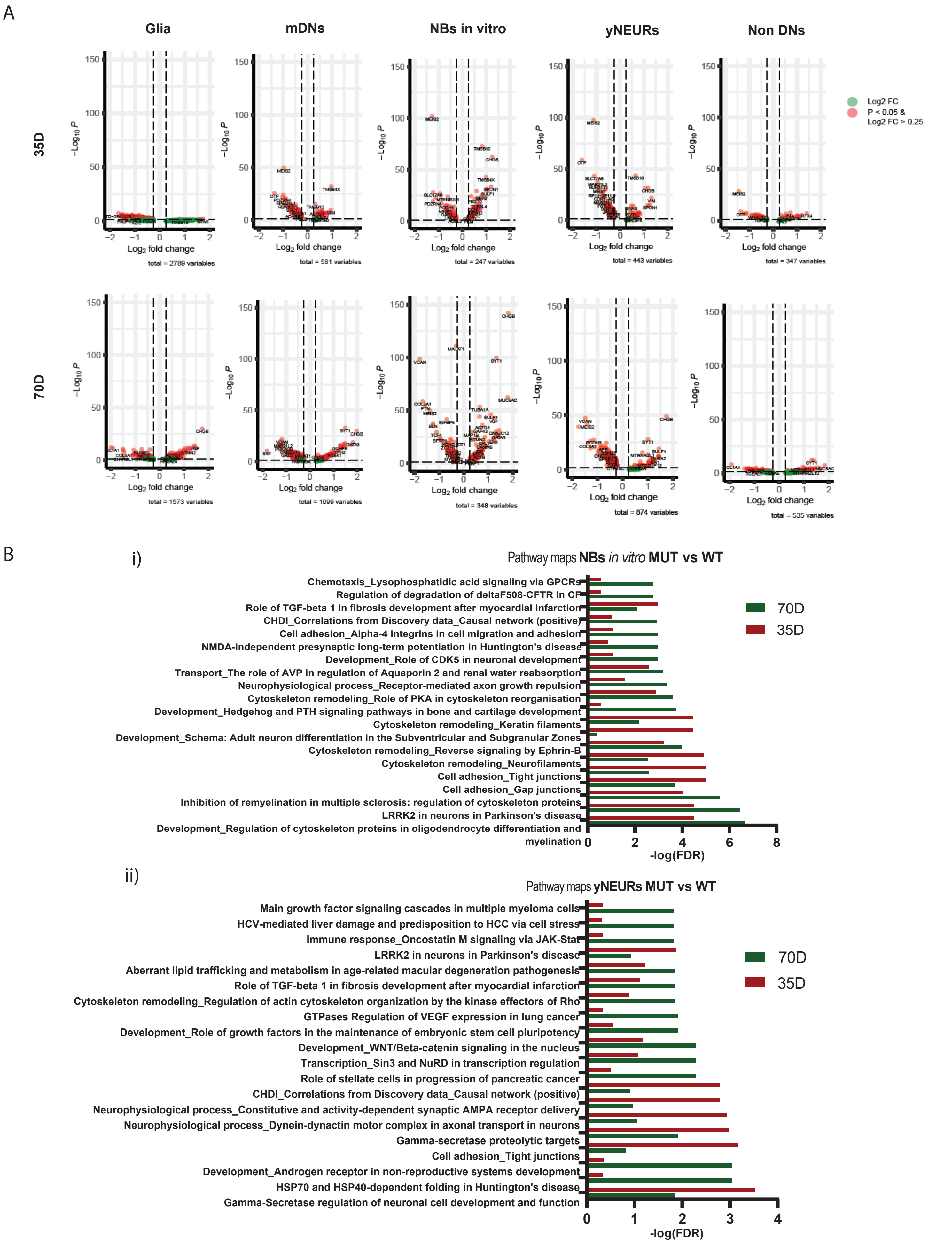

Figure S7. Differentially expressed gene analysis II. **A)** Volcano plots showing DEG fold changes in cell clusters between MUT and WT midbrain organoids. **B)** Pathway enrichment analysis in i) yNEURs and ii) NBs in vitro based on the DEGs in the respective cell clusters (adjusted p-value < 0.05).

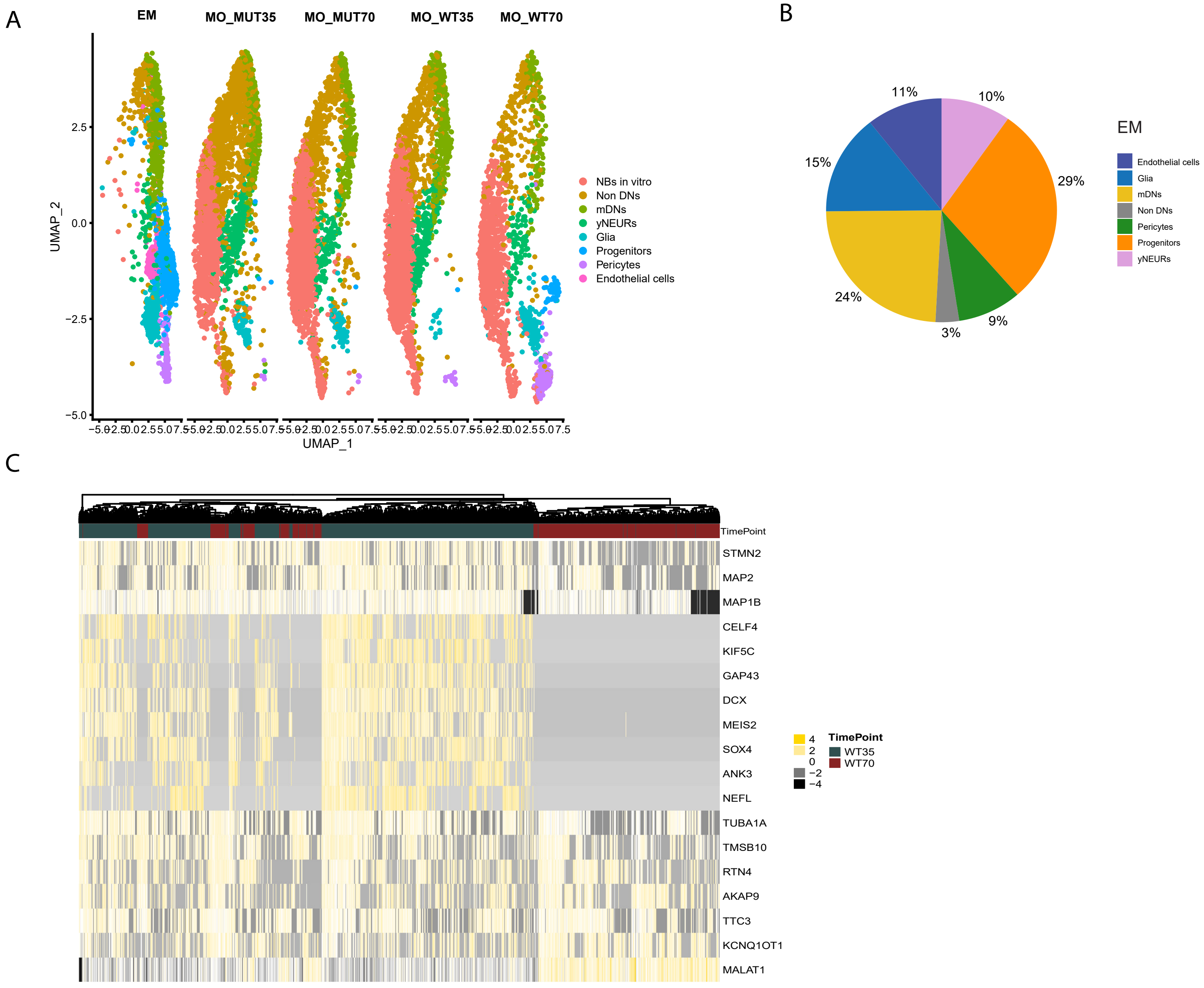

Figure S8. *LRRK2*-G2019S mutant midbrain organoids have a different cellular composition and altered developmental path. **A)** UMAP of integrated Seurat object of merged scRNAseq datasets of embryonic midbrain, and WT and MUT midbrain organoids 35 and 70 days of differentiation, colored by cell clusters and split by the dataset. **B)** Percentage of cell identities present in the embryonic midbrain (EM). **C)** Heatmap of genes with changed expression pattern in at least 50% of cells between WT35 and WT70 midbrain organoids.

Source of iPSCs	Mutation	Age at sampling	Sex	Corresponding midbrain organoid culture
The Wellcome Trust Sanger Institute, Cambridge, UK	-	55	Male	MO_WT35<br>MO_WT70
The Wellcome Trust Sanger Institute, Cambridge, UK	Introduced LRRK2 G2019S[1]	55	Male	MO_MUT35<br>MO_MUT70

Table S1. Cell lines used to generate midbrain floor plate neural progenitor cells and further the midbrain organoids for scRNAseq.

References of Supplemental Data

1. Qing, X., Walter, J., Jarazo, J., Arias-Fuenzalida, J., Hillje, A.L., and Schwamborn, J.C. (2017). CRISPR/Cas9 and piggyBac-mediated footprint-free LRRK2-G2019S knock-in reveals neuronal complexity phenotypes and α-Synuclein modulation in dopaminergic neurons. Stem Cell Res. *24*, 44–50.

2. Smits, L.M., Reinhardt, L., Reinhardt, P., Glatza, M., Monzel, A.S., Stanslowsky, N., Rosato-Siri, M.D., Zanon, A., Antony, P.M., Bellmann, J., et al. (2019). Modeling Parkinson's disease in midbrain-like organoids. Npj Park. Dis. *5*,.

3. Reinhardt, P., Glatza, M., Hemmer, K., Tsytsyura, Y., Thiel, C.S., Höing, S., Moritz, S., Parga, J.A., Wagner, L., Bruder, J.M., et al. (2013). Derivation and Expansion Using Only Small Molecules of Human Neural Progenitors for Neurodegenerative Disease Modeling. PLoS One *8*,.

4. Smits, L.M., Magni, S., Kinugawa, K., Grzyb, K., Luginbühl, J., Sabate-Soler, S., Bolognin, S., Shin, J.W., Mori, E., Skupin, A., et al. (2020). Single-cell transcriptomics reveals multiple neuronal cell types in human midbrain-specific organoids. Cell Tissue Res. *382*, 463–476.

5. La Manno, G., Gyllborg, D., Codeluppi, S., Nishimura, K., Salto, C., Zeisel, A., Borm, L.E., Stott, S.R.W., Toledo, E.M., Villaescusa, J.C., et al. (2016). Molecular Diversity of Midbrain Development in Mouse, Human, and Stem Cells. Cell 167, 566-580.e19.

6. Zhong, S., Zhang, S., Fan, X., Wu, Q., Yan, L., Dong, J., Zhang, H., Li, L., Sun, L., Pan, N., et al. (2018). A single-cell RNA-seq survey of the developmental landscape of the human prefrontal cortex. Nature 555, 524–528.

7. Trujillo, C.A., Gao, R., Negraes, P.D., Gu, J., Buchanan, J., Preissl, S., Wang, A., Wu, W., Haddad, G.G., Chaim, I.A., et al. (2019). Complex Oscillatory Waves Emerging from Cortical Organoids Model Early Human Brain Network Development. Cell Stem Cell 25, 558-569.e7.

8. Paisán-Ruíz, C., Jain, S., Evans, E.W., Gilks, W.P., Simón, J., Van Der Brug, M., De Munain, A.L., Aparicio, S., Gil, A.M., Khan, N., et al. (2004). Cloning of the gene containing mutations that cause PARK8-linked Parkinson's disease. Neuron 44, 595–600.

9. Ren, C., Ding, Y., Wei, S., Guan, L., Zhang, C., Ji, Y., Wang, F., Yin, S., and Yin, P.(2019). G2019S Variation in LRRK2: An Ideal Model for the Study of Parkinson's Disease? Front. Hum. Neurosci. 13, 1–6.
